# Adaptive evolution during the establishment of European avian‐like H1N1 influenza A virus in swine

**DOI:** 10.1111/eva.12536

**Published:** 2017-10-24

**Authors:** Udayan Joseph, Dhanasekaran Vijaykrishna, Gavin J.D. Smith, Yvonne C.F. Su

**Affiliations:** ^1^ Programme in Emerging Infectious Diseases Duke‐NUS Medical School Singapore; ^2^ Department of Microbiology Biomedicine Discovery Institute Monash University Melbourne Vic. Australia; ^3^ Duke Global Health Institute Duke University Durham NC USA

**Keywords:** cross‐species transmission, influenza A virus, natural selection, reassortment

## Abstract

An H1N1 subtype influenza A virus with all eight gene segments derived from wild birds (including mallards), ducks and chickens, caused severe disease outbreaks in swine populations in Europe beginning in 1979 and successfully adapted to form the European avian‐like swine (EA‐swine) influenza lineage. Genes of the EA‐swine lineage that are clearly segregated from its closest avian relatives continue to circulate in swine populations globally and represent a unique opportunity to study the adaptive process of an avian‐to‐mammalian cross‐species transmission. Here, we used a relaxed molecular clock model to test whether the EA‐swine virus originated through the introduction of a single avian ancestor as an entire genome, followed by an analysis of host‐specific selection pressures among different gene segments. Our data indicated independent introduction of gene segments via transmission of avian viruses into swine followed by reassortment events that occurred at least 1–4 years prior to the EA‐swine outbreak. All EA‐swine gene segments exhibit greater selection pressure than avian viruses, reflecting both adaptive pressures and relaxed selective constraints that are associated with host switching. Notably, we identified key amino acid mutations in the viral surface proteins (H1 and N1) that play a role in adaptation to new hosts. Following the establishment of EA‐swine lineage, we observed an increased frequency of intrasubtype reassortment of segments compared to the earlier strains that has been associated with adaptive amino acid replacements, disease severity and vaccine escape. Taken together, our study provides key insights into the adaptive changes in viral genomes following the transmission of avian influenza viruses to swine and the early establishment of the EA‐swine lineage.

## 1 INTRODUCTION

Influenza pandemics represent a significant threat to global public health. Four major pandemics have been recorded since the 1900s, occurring in 1918, 1957, 1968 and 2009 when influenza A viruses with genes from animal sources adapted to the human population, a process known as antigenic shift (Fraser et al., 2009; Garten et al., 2009; Kilbourne, 2006; Smith, Vijaykrishna, et al., 2009; Taubenberger, Hultin, & Morens, 2007; Webster, Bean, Gorman, Chambers, & Kawaoka, 1992). Phylogenetic analysis of viruses archived since the 1930s has shown that the H2N2/1957 and H3N2/1968 pandemic viruses resulted from reassortment of animal and circulating human viruses (Kawaoka, Krauss, & Webster, 1989; Schäffr et al., 1993; Scholtissek, Rohde, Von Hoyningen, & Rott, 1978). The H1N1/2009 pandemic virus emerged from swine that contained gene segments ultimately derived from previously circulating human and avian viruses, highlighting a key role of segmental reassortment of genes from multiple hosts for host adaption and pandemic emergence (Fraser et al., 2009; Garten et al., 2009; Smith, Vijaykrishna, et al., 2009).

The adaptation of an avian virus to mammalian hosts has been of great interest due to the pandemic potential of circulating low and highly pathogenic avian influenza (Herfst et al., 2012; Imai et al., 2012). While the origins of the 1918 virus is considered by some to have evolved directly from avian hosts (Taubenberger, Reid, Krafft, Bijwaard, & Fanning, 1997; Taubenberger et al., 2005; Worobey, Han, & Rambaut, 2014), phylogenetic studies using Bayesian molecular clock methods have shown that the origins of this virus may be staggered and that the role of the intermediate host, such as swine, may not be ruled out (Smith, Vijaykrishna, et al., 2009).

Swine influenza virus (SwIV) infection typically displays similar clinical symptoms that resemble infections in humans including high fever, nasal discharge and cough, although swine can also suffer from severe bronchitis and bronchiolitis that result in rapid breathing (tachypnea) and shortness of breath (dyspnoea) (Janke, 2013). Due to the presence of both human‐like (α‐2,6‐linked sialic acids) and avian‐like (α‐2,3‐linked sialic acid) virus receptors in the pig respiratory tract, swine have the ability to facilitate co‐infections of avian, swine and human influenza viruses generating novel reassortant viruses (Ito et al., 1998; Kida et al., 1994; Matrosovich et al., 2000; Rogers & Paulson, 1983; Rogers, Pritchett, Lane, & Paulson, 1983). This distinguishing feature has led to swine being referred to as “intermediate hosts” or “mixing vessels” that may serve as a potential reservoir in transmitting avian influenza viruses to humans (Ma, Kahn, & Richt, 2008; Scholtissek, 1990).

The Classical swine (CS) H1N1 viruses were the earliest recorded influenza virus in swine (Dowdle & Hattwick, 1977; Janke, 2013; Shope, 1931). Evolutionary analysis (Worobey et al., 2014) indicated that the CS HA‐H1 lineage directly descended from the human pandemic 1918 virus to form a stable lineage that has circulated for at least 80 years in swine. Reassortment of this lineage was first observed in 1998, when several severe outbreaks occurred in pig farms across North America (Webby et al., 2000; Zhou et al., 1999). These outbreaks were caused by the emergence of two distinctive H3N2 viruses: a double‐reassortant that contained CS H1N1 and human H3N2 virus genes, and a virus that contained the triple‐reassortant internal gene cassette (TRIG) that incorporated additional avian gene segments. The latter of the two viruses, which contains gene segments from the CS viruses (NS, NP and M), seasonal H3N2 viruses (HA, NA, PB1) and avian (PB2 and PA) viruses, eventually became more widespread in North America than the double‐reassortant virus and continues to circulate to date (Nelson et al., 2015; Webby et al., 2000). Other SwIV viruses that commonly circulate in North American swine populations include human‐like H1N1 and human‐like H3N2 and H1N2 viruses (Anderson et al., 2013; Vincent et al., 2014).

In contrast, the European avian‐like swine (EA‐swine) H1N1 virus was first detected in January 1979 in Belgium (Pensaert, Ottis, Vandeputte, Kaplan, & Bachmann, 1981). The virus spread to neighbouring countries such as Germany and France by late 1980 and eventually replaced CS viruses in Europe (Brown et al., 1997; Zell, Scholtissek, & Ludwig, 2013). Distinct from the CS lineage, the eight gene segments of EA‐swine lineage were derived from the avian influenza virus gene pool across Eurasia (Dunham et al., 2009; Lycett et al., 2012; Worobey et al., 2014; Zell et al., 2013). The EA‐swine virus subsequently spread to Asia, including Hong Kong and China, as early as 1993 (Vijaykrishna et al., 2011).

The three major SIV lineages (CS, TRIG and EA‐swine) differ in their ancestral origins, but more notably, they also vary in their rates of adaptive evolution (Bhatt et al., 2013): the EA‐swine virus exhibit faster rates than CS, reflecting that higher substitution rates are observed during host adaptation following cross‐species transmission. The EA‐swine H1N1 continued to circulate for many decades in Europe and donated genetic segments to form the following major lineages: EA‐swine H1N1, human‐like EA‐swine H3N2, human‐like EA‐swine H1N2 and pandemic H1N1 in humans. Furthermore, since 2009 at least 23 distinct genotypes (referred to as “A–W”) were identified in European swine (Watson et al., 2015).

Of significant importance to global human health is the emergence of 2009 H1N1 pandemic virus from swine to humans, as two genes (NA and M) were derived from EA‐swine viruses while the remaining segments originated from TRIG viruses (Garten et al., 2009; Smith, Vijaykrishna, et al., 2009). Furthermore, the HA of EA‐swine lineages is of particular concern as they preferentially bind to the human receptor, posing a potential threat of efficient transmission of EA‐swine from swine to humans (Yang et al., 2016). While recent attention has focused on understanding the evolutionary dynamics and migration patterns of SwIV, here we elucidate the evolutionary processes and adaptive changes of avian influenza viruses upon transmission to pigs. To do this, we applied a relaxed molecular clock to elucidate the molecular changes in adaptive evolution of the EA‐swine lineage at a genomic level. Identifying the key changes in cross‐species transmission will provide insight into the potential genetic factors that may be responsible for host adaptation and gain understanding into the patterns of adaptive evolution in establishing stable lineage descendants.

## 2 MATERIALS AND METHODS

### 2.1 Data set curation and maximum‐likelihood reconstruction

**Figure 1:**
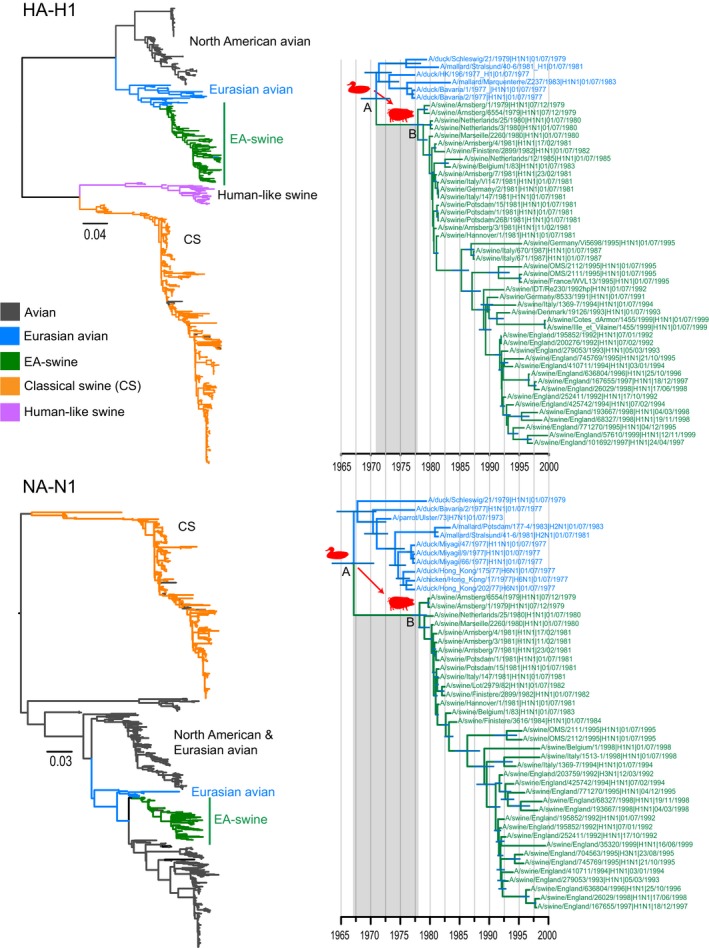
Evolution and divergence times of avian and swine HA‐H1 and NA‐N1 gene segments. Green branches denote the EA‐swine lineage; blue branches represent the closely related avian lineage from which the new swine lineage emerged; orange branches denote classical swine (CS); grey branches denote avian strains; and purple branches represent human‐like swine. The dated phylogenies were generated using 100 million MCMC generations, under a GTR+Γ substitution model, a coalescent‐based GMRF tree prior and an uncorrelated relaxed clock model. Node A denotes the mean TMRCA of the combined avian and EA‐swine, and node B represents the mean TMRCA of EA‐swine lineage only. Horizontal blue bars represent 95% highest posterior density (HPD) intervals, and shaded grey areas represent the period of cross‐species transmission. GMRF, Gaussian Markov random field; TMRCA, time to most recent common ancestor; EA‐swine, European avian‐like swine

Over 2000 nucleotide sequences of all H1NX (for HA), HXN1 (for NA) and HXNX (for all internal gene segments) isolated from avian and swine hosts before the year 2000 and their associated metadata were downloaded from the NCBI Influenza Virus Resource (http://www.ncbi.nlm.nih.gov/genomes/FLU/FLU.html). Sequence lengths of <500 bp were excluded from the analysis. For each gene segment, the large data sets were aligned using MAFFT (Katoh, Misawa, Kuma, & Miyata, 2002) followed by manual alignment. The sequences were trimmed to only include coding regions for subsequent analysis. Preliminary maximum‐likelihood (ML) analysis was conducted for large individual gene data sets (PB2, PB1, PA, HA, NP, NA, MP and NS) using RAxML v9.0 (Stamatakis, 2014). To focus on the adaptation and evolution of EA‐swine lineage, we used the resulting large ML trees (Figure 1; Figs S1–S8) to select the EA‐swine lineage and its closest avian lineage (denoted by blue branches in Figure 1 and Fig. S9). The latter included all available avian sequences that are exclusively form a sister group to the entire EA‐swine lineage. Therefore, distantly related avian lineages, with heterogeneous evolutionary rates that may affect phylogenetic analyses, were excluded in subsequent analyses. All duplicate viruses were manually removed from the data sets, and isolates with 100% identical residues were removed using the webserver of the program CD‐HIT (Huang, Niu, Gao, Fu, & Li, 2010; Li & Godzik, 2006). The program TempEst v1.5 (http://tree.bio.ed.ac.uk/software/tempest/) was also used to plot root‐to‐tip divergence times to remove any outliers from the sequence data sets as a possible result of mislabelled isolation dates. To account for uncertainty of isolation date, isolates were mid‐year‐rooted if the exact date of sampling was unknown. For each segment, the reduced data sets (comprising the EA‐swine and its closely related avian lineages) were used to reconstruct ML trees using a generalized time reversible nucleotide substitution model plus gamma distributed rates among sites (GTR+Γ) in PhyML v3.0 (Guindon et al., 2010).

### 2.2 Temporal phylogenies and estimation of TMRCAs

For each gene segment, the estimates of evolutionary rates and temporal phylogenies were performed in BEAST v1.8.2 (Drummond, Suchard, Xie, & Rambaut, 2012). An uncorrelated lognormal relaxed clock model within a Bayesian Markov chain Monte Carlo (MCMC) framework was used, with a Gaussian Markov random field coalescent tree prior. At least two independent MCMC runs of 100 million steps were performed and combined to ensure adequate sampling of all parameters, with a 10%–20% “burn‐in” removed in each run. The relevant statistics and values were parsed from these runs directly from the combined log files using the program Tracer v1.6 (http://tree.bio.ed.ac.uk/Tracer). Bayes factors (BF) for statistical support of differences between estimated time to most recent common ancestor (TMRCA) and nucleotide substitution rate values were calculated as described previously (Bahl, Vijaykrishna, Holmes, Smith, & Guan, 2009), where BF ≥ 150 indicates very strong support, 150 > BF ≥ 20 indicates strong support values, and 20 > BF ≥ 3 indicates supported values.

### 2.3 Selection analyses and ancestral node reconstruction

The estimates of the degree of natural selection were performed as previously described (Joseph et al., 2015). Briefly, the ratio of nonsynonymous to synonymous substitutions per codon (*d*_N_/*d*_S_ ratio) was estimated for each segment data set using the single‐likelihood ancestor counting (SLAC) method (Kosakovsky Pond & Frost, 2005) run through the Datamonkey webserver (Delport, Poon, Frost, & Kosakovsky Pond, 2010) with user‐supplied ML trees (as above). Specific amino acid sites of selection were determined using the Tdg09 program (Tamuri, Dos Reis, Hay, & Goldstein, 2009), with statistical cut‐offs of set at the false discovery rate (FDR) value of 0.20. Ancestral codon substitutions of nodes were determined using the baseml program of the PAML suite v4.7 (Yang, 2007) and transcribed onto trees generated with RAxML v8.1.6 (Stamatakis, 2014) using the treesub program (Tamuri, 2013), as described previously (Su et al., 2015).

### 2.4 Measurement of uracil content of avian and EA‐swine lineage

Differences in uracil content between EA‐swine and closely related avian lineages were performed similar to as previously described (Joseph et al., 2015) as it has been postulated that the uracil content of influenza A virus genomes tends to increase steadily in mammalian hosts over time (Rabadan, Levine, & Robins, 2006; Worobey et al., 2014). Briefly, uracil content values of the avian and EA‐swine gene segments were measured using PAUP* 4.0b10 (Swofford, 2003). Uracil content values were visualized and plotted using the R statistical software package (R Core Team 2013).

### 2.5 Detection of reassortment within the EA‐swine lineage

To identify the intralineage reassortment events in the EA‐swine virus lineage, we used the software Dendroscope v.3.0 (Huson & Scornavacca, 2012) to generate tanglegrams using the ML phylogenies. The ML phylogenies for individual gene segments of 38 EA‐swine isolates were reconstructed using IQ‐Tree v1.3.0 (Nguyen, Schmidt, von Haeseler, & Minh, 2015). The runs were performed using 10,000 ultrafast bootstrap replicates and automatic selection of best‐fit substitution model. The resulting ML phylogenies were rooted using the earliest EA‐swine strain (A/swine/Arnsberg/1979). To infer and visualize the reassortment events, auxiliary lines were then drawn between same set of virus isolates in the phylogenies of two gene segments (i.e., between HA and non‐HA phylogenies).

## 3 RESULTS

### 3.1 Large‐scale phylogenetic analysis of avian and swine H1N1 lineages in 1924–2000

**Figure 2:**
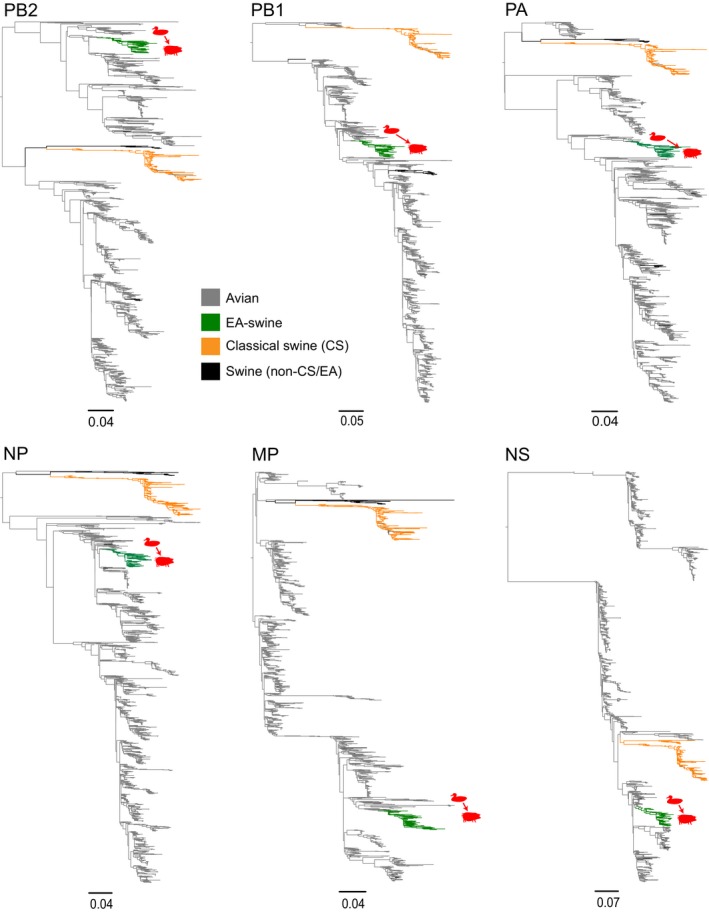
Evolution of avian and swine internal gene segments. Green branches denote the EA‐swine lineage; orange branches denote classical swine (CS); and grey branches denote avian strains. Red arrows indicate the avian‐to‐swine cross‐species transmission events for each gene. EA‐swine, European avian‐like swine

Maximum‐likelihood phylogenies of all eight individual gene segments (Figures 1 and 2; Figs S1–S8) show that the EA‐swine lineages (denoted by green branches) were monophyletic and clearly distinct to the CS swine lineage (denoted by orange branches). In all eight gene segments, the EA‐swine lineages were derived from various Eurasian avian lineages. The most closely related avian gene segments were from different subtypes and were predominantly found in aquatic birds across Europe. For instance, the EA‐swine PB2 was most closely related to genes from duck H2N3 (Germany) and turkey H5N2 (United Kingdom), while the NS was most closely related to genes from duck H1N1 (France) and H2N3, H7N7 (Germany). A few avian H1 strains, isolated from domestic turkey in Europe, nested within the EA‐swine lineage in all eight genes although without apparent evidence of sustained transmission, suggesting incidental infection arising due to close proximity of high‐density turkey and swine populations in Europe during that period (Bonfante et al., 2016). Taken together, these results indicate that the EA‐swine lineage virus is unlikely to have resulted from a single avian‐to‐swine transmission.

### 3.2 Divergence times and natural selection of the EA‐swine lineage and its closest avian relatives

The availability of genome sequences from the EA‐swine lineage since 1979 and data from parallel surveillance in aquatic bird surveillance in Europe that contained the closest avian relatives (Figures 1 and 2) enabled us to estimate the time of divergence of the EA‐swine lineage. Dated phylogenies of all eight genes (Figure 1: HA and NA; Fig. S9: six internal genes) showed that the TMRCAs of the EA‐swine and the closest avian lineages (combined avian and EA‐swine lineage in Table S1) ranged from 1962 for the MP gene to 1974 for the NS gene, indicating an unsampled diversity of 4 to 17 years of these gene segments during which cross‐species transmission may have occurred. However, it is not conclusive if these avian gene segments were introduced into swine as early as 17 years before first EA‐swine detection from 1979 onwards as influenza surveillance in wild bird populations was not widespread during this period.

**Figure 3:**
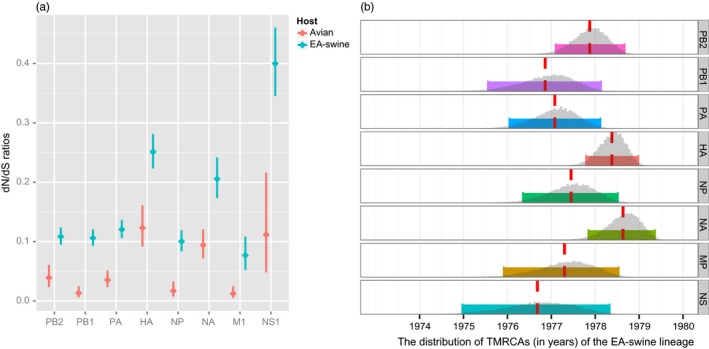
Selection pressure and estimated time of the most recent common ancestors (TMRCAs) of the EA‐swine lineage. (a) Average *d*_N_/*d*_S_ ratios (with 95% confidence intervals) of proteins compared between avian (pink) and swine (blue) lineages. Only ratios of M1 and NS1 proteins depicted as representation of the in‐frame translation of the MP and NS segments, respectively. (b) Dotted red lines represent the mean TMRCAs, solid coloured bars denote the 95% HPD intervals, and the grey histograms represent the TMRCA distribution across 18,000 sampled Bayesian MCMC trees. EA‐swine, European avian‐like swine; MCMC, Markov chain Monte Carlo

To determine the TMRCAs of the EA‐swine viruses, we performed independent analyses of the EA‐swine lineage. The mean nucleotide substitution rates of EA‐swine lineage varied in different gene segments (Table 1), ranging from 2.31–4.20 × 10^−3^ substitutions per site per year (subs site^−1^ year^−1^). The HA‐H1 and NA‐N1 segments exhibited higher substitution rates (4.20 × 10^−3^ and 3.50 × 10^−3^ subs site^−1^ year^−1^, respectively) than the internal segments. For each gene segment, the mean TMRCAs of the EA‐swine lineages were estimated as between September 1976 and August 1978 (Figure 3b, Table 1, Fig. S10). The PB1 and NS genes estimated TMRCA were older than other genes, at 1976.86 (95% HPD: 1975.45–1978.02) and 1976.68 (95% HPD: 1974.96–1978.32), respectively. This suggests that the avian PB1 and NS genes may have been introduced into the new swine host populations as early as December 1974. In contrast, the HA and NA genes were introduced into the swine populations later, at 1978.38 and 1978.63, respectively. More notably, our BF test demonstrated the differences in TMRCA between segments were statistically significant (Table S2): all internal segments were older (i.e., circulating longer in swine) than the HA and NA segments. Among the internal gene segments, the NS was significantly older than PB2, PB1 and NP, whereas the PB2 was younger than the PB1 and PA, and the NP as significantly younger than the PB1. It is therefore possible that these gene segments were independently introduced from birds to pigs through a series of reassortment events and circulated undetected during this period.

**Table 1:** Times to most common recent ancestor (TMRCA) and nucleotide substitution rates of all eight segments of the EA‐swine lineage viruses (Fig. S10)

Gene segment	TMRCA of EA‐swine (year)			Nucleotide substitution rate of EA‐swine (subs site^−1^ year^−1^)		
	Mean	Lower 95% HPD	Upper 95% HPD	Mean	Lower 95% HPD	Upper 95% HPD
PB2	1977.88 (19 November 1977)	1977.09	1978.66	3.06E‐03	2.73E‐03	3.40E‐03
PB1	1976.86 (12 November 1976)	1975.45	1978.02	2.65E‐03	2.24E‐03	3.06E‐03
PA	1977.08 (30 January 1977)	1976.03	1978.11	2.89E‐03	2.55E‐03	3.23E‐03
HA	1978.38 (20 May 1978)	1977.78	1978.97	4.20E‐03	3.67E‐03	4.72E‐03
NP	1977.45 (14 June 1977)	1976.34	1978.50	2.47E‐03	2.11E‐03	2.85E‐03
NA	1978.63 (19 August 1978)	1977.83	1979.35	3.50E‐03	3.01E‐03	4.02E‐03
MP	1977.30 (21 April 1977)	1975.91	1978.52	2.31E‐03	1.83E‐03	2.82E‐03
NS	1976.68 (06 September 1976)	1974.96	1978.32	2.41E‐03	2.91E‐03	3.44E‐03

EA‐swine, European avian‐like swine; HPD, highest posterior density.

We further estimated and compared the average *d*_N_/*d*_S_ ratios between the avian and EA‐swine lineages across all eight gene segments (Figure 3a, Table 2). The SLAC analysis revealed that the EA‐swine lineage exhibited a markedly greater level of selection pressures (i.e., higher *d*_N_/*d*_S_) in most genes (with exception to alternatively spliced M2 and NS2 genes) than the avian lineages. More specifically, the HA, NA and NS1 genes displayed more adaptive pressures than the other segments. Greater *d*_N_/*d*_S_ in the NS1 gene could be indicative of antagonizing host type I interferon innate immune response (Wei, Chen, Lin, & Pan, 2014). However, it should also be noted that the mean *d*_N_/*d*_S_ value (0.40; 95% HPD 0.35–0.46) of the NS1 in our analysis is comparable with other a previous study of EA viruses from swine in Hong Kong (0.30; 95% HPD 0.23–0.39) that only included viruses sampled from 2001 to 2010 (Vijaykrishna et al., 2011). These results are therefore consistent with previous observations.

**Table 2:** Node reconstruction and selection pressures of EA‐swine lineage viruses

Gene segment	Ancestral node substitutions	Tdg09 (FDR ≤ 0.20)^a^	Data set	SLAC		
		# Significant residues (Site number)		Mean	95% CI	
					Lower	Upper
PB2	R251K, F446L	7 (21, 251*, 338, 400, 624, 649, 701)	Avian	0.0391	0.0233	0.0608
			Swine	0.1084	0.0945	0.1236
PB1	R584H, A587T	0	Avian	0.0132	0.0061	0.0247
			Swine	0.1061	0.0928	0.1206
PA	I62V, E258D, V387I, E399K	7 (262, 387*, 399*, 400, 684, 688, 712)	Avian	0.0353	0.0232	0.0511
			Swine	0.1205	0.1059	0.1363
HA	K130R, S138N, T169I, A202T, E204D, G239E, T393S, N416D, R419K, S457F	33 (3, 4, 36, 60, 113, 130*, 154, 155, 159, 169*, 173, 204*, 224, 239*, 277, 279, 284, 293, 306, 312, 328, 338, 387, 393*, 405, 409, 416*, 419*, 444, 457*, 465, 490, 502)	Avian	0.1232	0.0917	0.1611
			Swine	0.2511	0.2232	0.2814
NP	V190I	8 (48, 84, 98, 99, 190*, 284, 351, 384)	Avian	0.017	0.0073	0.0329
			Swine	0.1002	0.0837	0.1188
NA	V16I, V17I, T40I, I53V, I106V, A166V, V321I, G354D	31 (16*, 17*, 34, 40*, 46, 73, 75, 81, 82, 84, 94, 105, 106*, 111, 149, 166*, 234, 260, 262, 265, 285, 313, 314, 344, 354*, 374, 385, 386, 389, 395, 454)	Avian	0.0941	0.0717	0.1208
			Swine	0.2056	0.1732	0.2418
M1	None	4 (15, 116, 214, 248)	Avian	0.0124	0.0049	0.0251
			Swine	0.077	0.0522	0.1085
M2^b^	None	1 (18)	Avian	0.6904	0.418	1.0621
			Swine	0.7086	0.5227	0.9347
NS1	Q25R, E66K, T197A, L214F	9 (4, 129, 152, 158, 200, 208, 213, 214*, 227)	Avian	0.1118	0.048	0.2163
			Swine	0.4	0.3451	0.4605
NS2^b^	None	8 (4, 26, 49, 50, 52, 56, 60, 70)	Avian	0.1418	0.044	0.3298
			Swine	0.2774	0.2073	0.3617

EA‐swine, European avian‐like swine.

a False discovery rate (FDR). Sites with asterisks indicate residues undergoing mutation in the branch of interest (i.e., avian to EA‐swine lineage).

b Selection calculations for alternatively spliced proteins must be interpreted with caution, as selection pressure acts on the main segment/protein reading frame.

In addition, we reconstructed the ancestral nonsynonymous mutations occurring along the branches that were involved in the host switch from avian to swine. Our results identified at least 30 amino acid mutations across all eight gene segments, with a larger proportion of mutations occurring in the surface HA and NA genes (10 and eight nonsynonymous substitutions, respectively) (Table 2). Greater HA and NA mutations may also be correlated with higher mean substitutions rates that are observed in both genes (see above).

**Figure 4:**
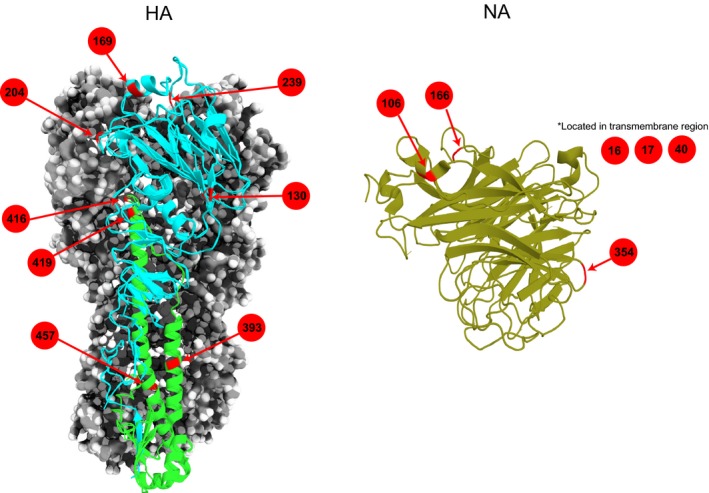
Mapping of the significant amino acid sites on the 3D structures of HA and NA molecules. Structural images were generated in MacPyMOL (The PyMOL Molecular Graphics System, version 1.8.0.3, Schrödinger, LLC): HA trimer [PDB accession code 4F3Z], and NA monomer crystal structures [PDB accession codes 4B7N]. Red arrows with numbers denote the amino acid positions that are predicted to be significant in adaptation to swine hosts

We also applied the Tdg09 method and designated the host‐specific lineages to estimate the site‐specific selection of amino acids that are likely responsible for the host switching. The results indicated that 19 of the 30 nonsynonymous mutations that occurred across all eight segments with a statistical cut‐off of FDR ≤ 0.20 (Table 2), suggesting that the remaining residue changes are likely founder mutations or a by‐product of relaxed selection occurring in the establishment of a new host species (Su et al., 2015; Wertheim, Murrell, Smith, Kosakovsky Pond, & Scheffler, 2015). The surface HA and NA proteins showed a greater number of significant amino acid mutations compared to the internal gene segments. Of these statistically significant residues, mutations in the HA genes may have major implications in the cross‐species transmission of the avian H1 segment into swine host (Figure 4): amino acid positions at 169, 204 and 209 (155, 190 and 225 in H3 numbering) situated on the HA globular head were deemed to be responsible for mediating host receptor switching between the use of α‐2,3 and α‐2,6 sialic acid residues (Glaser et al., 2005; Lin et al., 2012; Stevens et al., 2006), and may be involved in facilitating the adaptation of avian influenza virus to the new swine host. Other significant mutations were found in the conserved stalk region of the HA2 protein that occurred at positions 416, 419 and 457 (HA2–72, 75 and 113 in H3 numbering). The mutations at these sites in the swine have proven to play an important role in altering conformational HA stability and increasing optimal membrane fusion pH of the H1N1 HA protein, and thus are important adaptive mutations in avian‐to‐swine cross‐species transmission (Baumann, Mounogou Kouassi, Foni, Klenk, & Matrosovich, 2015). To a lesser extent, other amino acid mutations at 130 and 393 (HA–120 and HA2–49 in H3 numbering) have been reported to have implications in mediating antibody binding (Ekiert et al., 2009; Zhu et al., 2013).

For the host jump from avian NA‐N1 to swine, we identified amino acid positions 16 and 17 that are located in the transmembrane region of the NA protein (Figure 4). Their functions were reported to anchor the protein to membrane as well as be part of the signal peptide that permits transport of the protein across the endoplasmic membrane (Air, 2012). Therefore, adaptive mutations in this region may have altered the stability of the NA protein attachment to the membrane for better stability in the new swine hosts. In contrast, fewer amino acid mutations are present in the internal gene segments of EA‐swine virus. Of note, mutation at the PB2‐701 has been reported to be correlated with replication efficiency and these play a role in host adaptation of avian influenza viruses to mammalian hosts (Czudai‐Matwich, Otte, Matrosovich, Gabriel, & Klenk, 2014; Dunham et al., 2009; Hayashi, Wills, Bussey, & Takimoto, 2015).

### 3.3 Patterns of uracil content variation in avian and EA‐swine lineages

**Figure 5:**
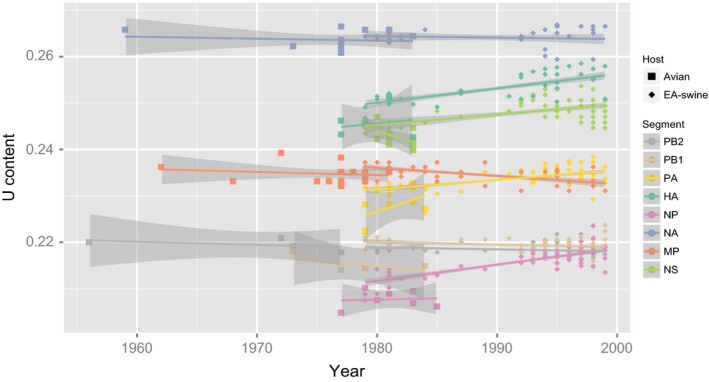
Uracil content of each gene segments in avian and EA‐swine viruses. Different colour dots represent different gene segments. The avian isolates are denoted by the solid square symbol, whereas the swine isolates are indicated by the solid diamond symbol. Coloured lines are the best‐fit regression lines, and the shaded grey areas represent the 95% confidence intervals. EA‐swine, European avian‐like swine

To understand the potential role of changes in uracil content in the adaptive evolution of the EA‐swine lineage, we evaluated and compared uracil content for all EA‐swine segments in comparison with avian viruses. Within the avian lineage, no observable changes in uracil contents for most genes, except for a slight decrease in NS gene, were observed, while the reverse was found in the PA gene (Figure 5). In contrast, we observed differential uracil patterns in the swine segments from 1980 to 2000: the HA, PA, NS and NP segments underwent a gradual increase in uracil content, suggesting greater uracil content in mammals compared to avian species may be required for the adaptation of new hosts. However, the swine PB2, PB1 and MP displayed a slight decrease in uracil content, whereas the swine NA did not undergo any apparent change uracil content.

### 3.4 Reassortment within the EA‐swine lineage

The extent of reassortment among the EA‐swine lineage was assessed by comparing topological congruence between swine gene segments (Figs S11–S19). The HA tanglegram was rather incongruent with that of the internal genes, particularly in the PB1, NP, MP and NS genes. In addition, the more recent EA‐swine strains have undergone more intense intrasubtype reassortment compared with the earlier strains, and coincided with the virus diversification that began in the late 1980s.

## 4 DISCUSSION

The EA‐swine virus successfully replaced the CS virus in Europe, as the infection in swine has been shown to enhance greater viral replication and induce prolonged virus shedding with higher viral loads in comparison with the CS swine virus, and the pigs were found to have low level of cross‐reactive antibodies to EA‐swine virus (Vijaykrishna et al., 2011). Furthermore, faster adaptive evolution was clearly observed in the EA‐swine lineage compared to the CS lineage, which correlated with the establishment in the new swine host (Bhatt et al., 2013).

In the present study, we reconstructed large phylogenies of avian and swine viruses from Eurasia and North America. All individual gene phylogenies indicated the EA‐swine virus formed a distinct lineage that is segregated from its closest avian relatives as well as CS. We further evaluated the selection pressure of this monophyletic EA‐swine lineage. Our data demonstrated marked variation in the selection pressure between the avian and EA‐swine viruses: the EA‐swine virus experienced greater *d*_N_/*d*_S_ ratios (0.10–0.28) than the avian virus (generally < 0.5) in most segments, especially in the swine HA, NA and NS segments. Notably, the M2 of both avian (*d*_N_/*d*_S_ of 0.69) and EA‐swine (*d*_N_/*d*_S_ of 0.71) viruses had exceptionally greater relaxed selection pressure in comparison with other segments. The differences in selection pressures in the M1 and M2 could be associated with host immune response and tropism (Furuse, Suzuki, Kamigaki, & Oshitani, 2009).

Independent estimation of ancestral nonsynonymous mutations of EA‐swine lineage revealed that adaptive mutations were likely involved with the establishment of the EA‐swine lineage. Strikingly, estimates of site‐specific selection indicated 30 nonsynonymous mutations across most segments, probably because of relaxed selection or founder effects. The EA‐swine HA and NA segments have undergone greater amino acid mutations than other segments, which also correspond with faster rates of nucleotide substitutions in the surface proteins. Out of 30 sites, 19 sites are significant mutations that may play a crucial role in host switching of influenza A viruses from avian to swine hosts. However, the adaptive roles of most amino acid changes are still largely unknown, and further experimental characterization of these identified sites of substitution is needed to understand the adaptation of avian influenza A viruses into mammalian hosts. It is noteworthy that the swine MP gene did not seem to undergo ancestral nonsynonymous mutations. This suggests that the gene may not necessarily require significant adaptive change to achieve cross‐species transfer of this gene.

The mean TMRCAs of all gene segments of the monophyletic avian and EA‐swine lineage ranged from 1962 to 1974, which is consistent with previous age estimations (Dunham et al., 2009; Krumbholz et al., 2014). This also represents 4–17 years of unsampled diversity prior to the outbreak during which cross‐species transmission may have occurred. The mean TMRCAs of EA‐swine lineage gene segments, when calculated alone, ranged from 1976 to 1978 with the HA and NA segment estimates significantly younger than the internal segments, at 1978.38 (May 1978) and 1978.63 (August 1978), respectively. Among the internal segments, the BF test also demonstrated the NS was markedly older than PB2, PB1 and NP segments, whereas the PB2 was younger than PB1, PA and NP segments. The previous hypothesis that the EA‐swine virus was derived from an avian source in the absence of reassortment (Pensaert et al., 1981; Schultz, Fitch, Ludwig, Mandler, & Scholtissek, 1991; Vincent et al., 2014) is not supported by our age estimates, which differ significantly between gene segments. If the EA‐swine virus had resulted from a single transmission of an entire virus from birds to pigs, then there would not be a statistical difference in the ages of the EA genes in pigs (Guan et al., 2010; Smith, Bahl, et al., 2009). Moreover, a different group of virus subtypes from wild and domestic birds were ancestral to each gene segment of the EA‐swine lineage viruses, reflecting the acquisition of gene segments in swine from multiple sources. Taken together, our results highlight that the establishment of the EA‐swine H1N1 virus was achieved through the independent introduction of avian gene segments via transmission of viruses into swine followed by reassortment events that occurred at least 1–4 years prior to the EA‐swine outbreak.

Following the establishment of EA‐swine lineage, we observed intrasubtype reassortment of segments occurred more frequently in subsequent EA‐swine strains than the earlier strains. Frequent intrasubtype reassortment as in the case of H3N2 has been shown to be associated with adaptive amino acid replacements, disease severity and vaccine escape (Berry et al., 2016; Neverov, Lezhnina, Kondrashov, & Bazykin, 2014). We speculate this could be due to the close geographical proximity of farmyards within the Eurasian region and increased reassortment could facilitate sustained swine‐to‐swine transmission in Eurasian swine. In addition, EA‐swine virus has been reported to experience greater rate of intersubtype reassortment with H1N2 virus within swine herds, with an average of one reassortment every 2–3 years (Lycett et al., 2012).

In summary, some notable adaptation phenomena could be indicative of cross‐species transmission of influenza A viruses: internal viral proteins began to alter and appear to be better adapted to new hosts, followed by the introduction of the HA and NA surface proteins with greater levels of selective pressure. New reassortant viruses are then capable of efficient replication and sustained transmission in the new host; this is similarly observed in the H2N2 pandemic (Joseph et al., 2015) and H1N1 pandemic (Su et al., 2015). There is little doubt that influenza reassortment and the establishment of novel gene constellations in new host species have been repeatedly shown to cause panzootics and pandemics that could threaten global public health and cause significant economic losses. More crucially, the HA of EA‐swine virus is of pandemic risk as it is antigenically different to currently circulating human H1 viruses in humans, and therefore, there would be little population immunity in humans against viruses carrying this HA. While the enhanced efforts to limit the mixing of different livestock have been implemented by increasing biosecurity measures on farms, continued global surveillance of swine and human populations is paramount in identifying and preventing zoonotic infections of humans.

## DATA ARCHIVING STATEMENT

Genetic sequences used in the analyses are available from GenBank and accession numbers are provided in Figs S1–S8.
