# Supplementary material for: Adaptive evolution during the establishment of European avian‐like H1N1 influenza A virus in swine
Source: Evol Appl. 2017 Oct 24;11(4):534–46. doi: 10.1111/eva.12536 (PMC5891058; doi:10.1111/eva.12536)
Supplement: Supplementary file 1 [file EVA-11-534-s001.pdf]

Supplementary Table 1. Times to most common recent ancestor (TMRCA) and nucleotide substitution rates of all eight segments of the combined EA-swine and closely related Eurasian avian influenza viruses (Figure 1 and Supplementary Fig 9).

Gene segment	TMRCA of combined EA-swine and closely related avian virues (node A)			TMRCA of EA-swine (node B)			Nucleotide substitution rate (subs/site/year)		
	Mean (year)	Upper 95% HPD	Lower 95% HPD	Mean (year)	Upper 95% HPD	Lower 95% HPD	Mean	Lower 95% HPD	Upper 95% HPD
PB2	1966.98	1964.86	1969.03	1976.95	1974.58	1978.65	2.01E-03	2.27E-03	2.53E-03
PB1	1969.31	1966.05	1971.92	1976.06	1974.14	1977.67	2.30E-03	2.67E-03	3.03E-03
PA	1971.94	1968.91	1974.74	1975.76	1973.40	1977.94	2.68E-03	3.14E-03	3.62E-03
HA	1970.93	1968.36	1973.26	1977.87	1976.72	1978.95	3.48E-03	4.10E-03	4.72E-03
NP	1971.31	1968.76	1973.62	1975.68	1972.66	1978.12	2.08E-03	2.53E-03	2.96E-03
NA	1967.15	1963.41	1970.58	1977.72	1975.26	1979.44	1.83E-03	2.24E-03	2.66E-03
MP	1962.02	1958.07	1965.71	1975.59	1971.18	1978.74	1.31E-03	1.66E-03	2.04E-03
NS	1974.84	1972.91	1976.44	1975.87	1973.94	1977.74	2.10E-03	2.54E-03	3.00E-03

Supplementary Table 2. Bayes factor values for the mean TMRCAs of EA-swine lineage viruses.

!

!

!

Scenario	Bayes Factor
HA older than NA	9.60
HA older than PB1	0.00
HA older than PB2	0.00
HA older than PA	0.00
HA older than MP	0.02
HA older than NS	0.00
HA older than NP	0.01
NA older than HA	1.64
NA older than PB1	0.01
NA older than PB2	0.10
NA older than PA	0.01
NA older than MP	0.04
NA older than NS	0.27
NA older than NP	0.02
PB1 older than HA	528.10
PB1 older than NA	150.96
PB1 older than PB2	13.62
PB1 older than PA	1.56
PB1 older than MP	2.17
PB1 older than NS	0.01
PB1 older than NP	16.68
PB2 older than HA	436.74
PB2 older than NA	10.22
PB2 older than PB1	0.07
PB2 older than PA	0.23
PB2 older than MP	0.48
PB2 older than NS	0.07
PB2 older than NP	1.39

!

Scenario	Bayes Factor
PA older than HA	160.45
PA older than NA	174.41
PA older than PB1	0.64
PA older than PB2	4.28
PA older than MP	1.63
PA older than NS	0.53
PA older than NP	1.30
MP older than HA	53.06
MP older than NA	26.38
MP older than PB1	0.46
MP older than PB2	2.09
MP older than PA	0.61
MP older than NS	0.41
MP older than NP	1.26
NS older than HA	271.05
NS older than NA	1500.56
NS older than PB1	99.95
NS older than PB2	13.77
NS older than PA	1.90
NS older than MP	2.44
NS older than NP	20.61
NP older than HA	123.17
NP older than NA	41.81
NP older than PB1	0.06
NP older than PB2	2.12
NP older than PA	0.77
NP older than MP	1.26
NP older than NS	0.05

!

!

Legend	
Bayes Factor	Level of Support
3–19	Supported
20–150	Strongly Supported
>150	Very Strongly Supported

Figure captions for Supplementary Figures

Supplementary Figure 1. Maximum likelihood phylogeny of avian and swine influenza PB2 segments. Green branches denote the EA-swine lineage; orange branches denote classical swine (CS); grey branches denote avian strains.

Supplementary Figure 2. Maximum likelihood phylogeny of avian and swine influenza PB1 segments. Green branches denote the EA-swine lineage; orange branches denote classical swine (CS); grey branches denote avian strains.

Supplementary Figure 3. Maximum likelihood phylogeny of avian and swine influenza PA segments. Green branches denote the EA-swine lineage; orange branches denote classical swine (CS); grey branches denote avian strains.

Supplementary Figure 4. Maximum likelihood phylogeny of avian and swine influenza HA-H1 segments. Green branches denote the EA-swine lineage; orange branches denote classical swine (CS); grey branches denote avian strains.

Supplementary Figure 5. Maximum likelihood phylogeny of avian and swine NP influenza segments. Green branches denote the EA-swine lineage; orange branches denote classical swine (CS); grey branches denote avian strains.

Supplementary Figure 6. Maximum likelihood phylogeny of avian and swine influenza NA-N1 segments. Green branches denote the EA-swine lineage; orange branches denote classical swine (CS); grey branches denote avian strains.

Supplementary Figure 7. Maximum likelihood phylogeny of avian and swine influenza MP segments. Green branches denote the EA-swine lineage; orange branches denote classical swine (CS); grey branches denote avian strains.

Supplementary Figure 8. Maximum likelihood phylogeny of avian and swine influenza NS segments. Green branches denote the EA-swine lineage; orange branches denote classical swine (CS); grey branches denote avian strains.

Supplementary Figure 9. Dated phylogenies of the internal genes of the combined EA-swine and closely related avian influenza viruses. Green branches denote the EA-swine lineage and blue branches denote avian strains.

Supplementary Figure 10. Dated phylogenies of the HA-H1, NA-N1 and the internal genes of the EA-swine influenza virus.

Supplementary Figure 11. Tanglegram of EA-swine HA and PB2 segments.

Supplementary Figure 12. Tanglegram of EA-swine HA and PB1 segments.

Supplementary Figure 13. Tanglegram of EA-swine HA and PA segments.

Supplementary Figure 14. Tanglegram of EA-swine HA and NP segments.

Supplementary Figure 15. Tanglegram of EA-swine HA and NA segments.

Supplementary Figure 16. Tanglegram of EA-swine HA and M1 segments.

Supplementary Figure 17. Tanglegram of EA-swine HA and M2 segments.

Supplementary Figure 18. Tanglegram of EA-swine HA and NS1 segments.

Supplementary Figure 19. Tanglegram of EA-swine HA and NS2 segments.

PB2 (2745 sequences)

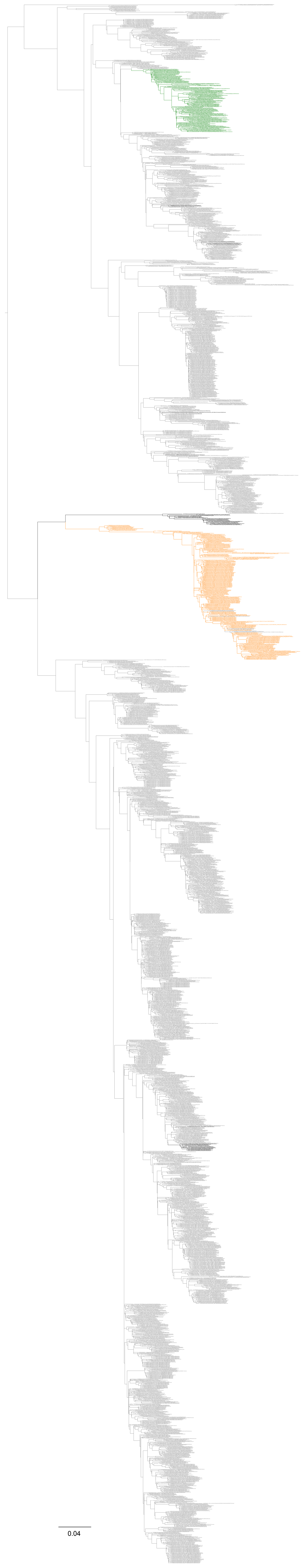

PB1 (2779 sequences)

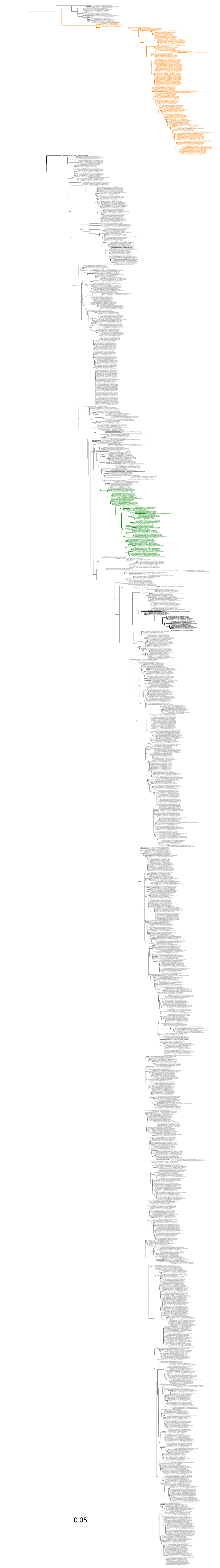

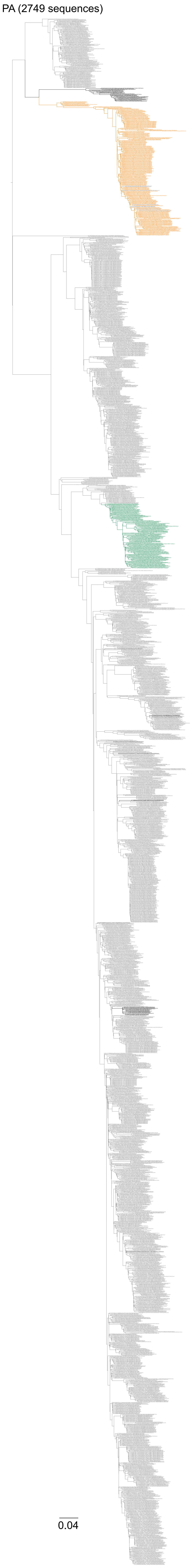
PA (2749 sequences)
0.04

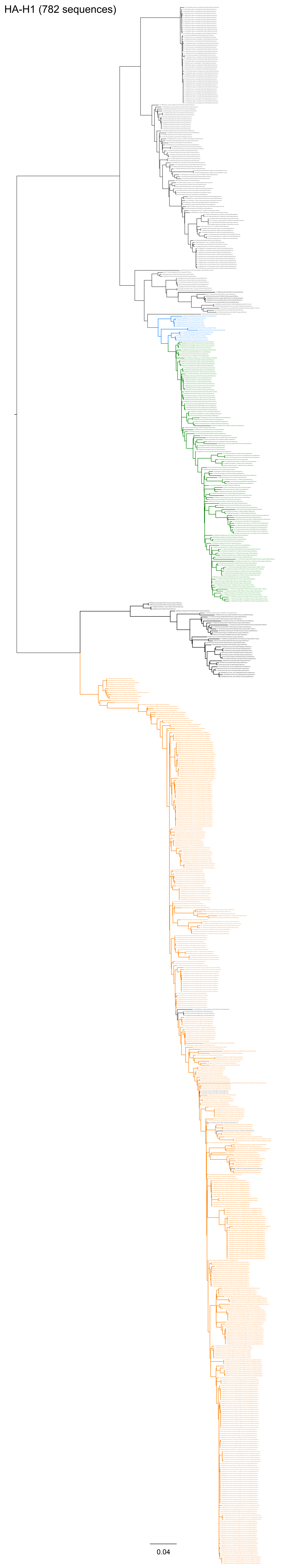
HA-H1 (782 sequences)
0.04

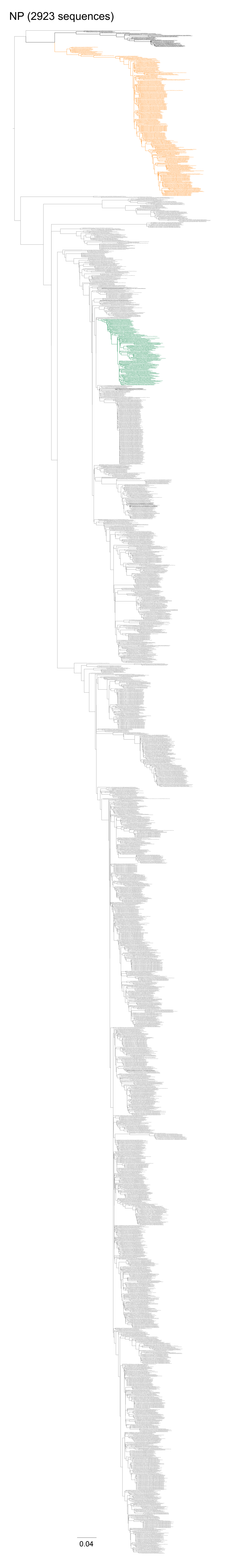
NP (2923 sequences)
0.04

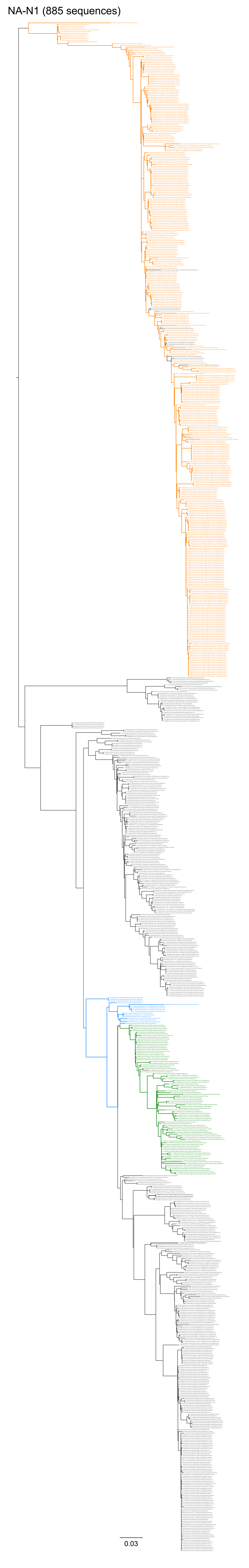
NA-N1 (885 sequences)
0.03

MP (3199 sequences)

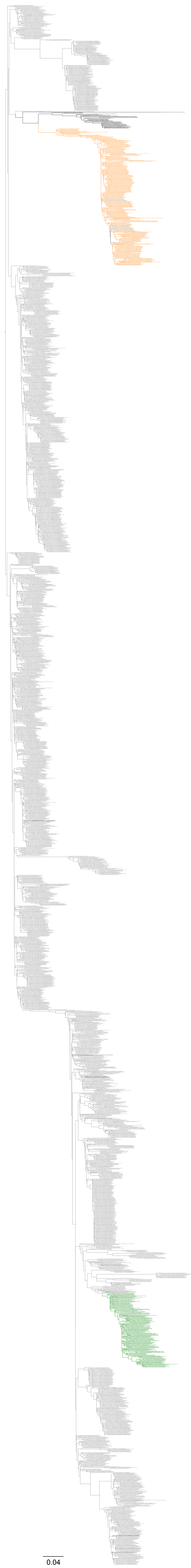

NS (3027 sequences)

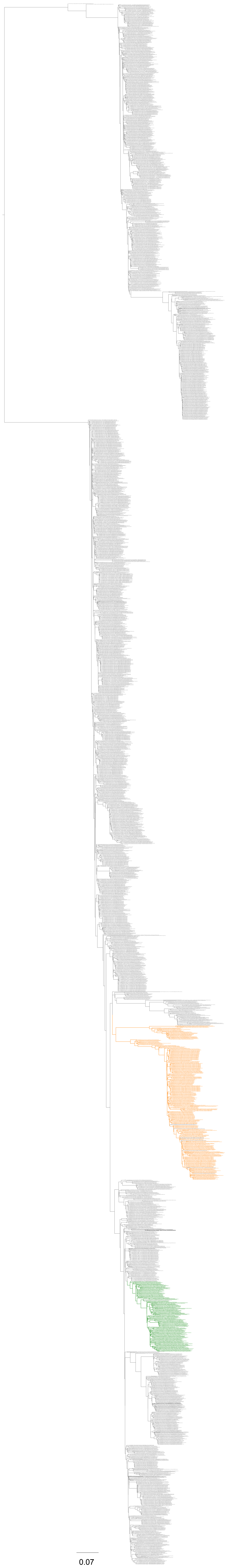

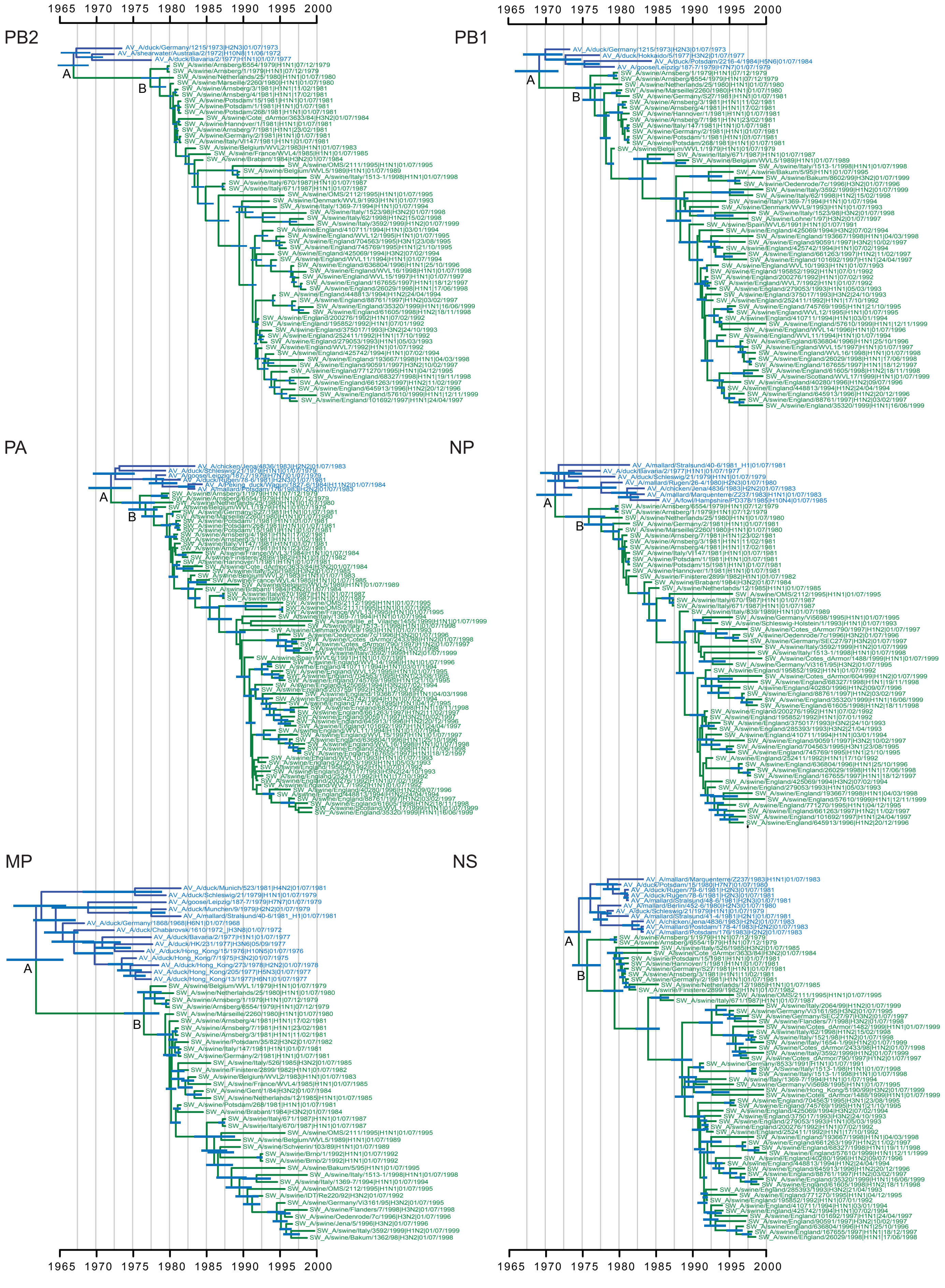

PB2
PB1
PA
NP
MP
NS
1965 1970 1975 1980 1985 1990 1995 2000
A
B

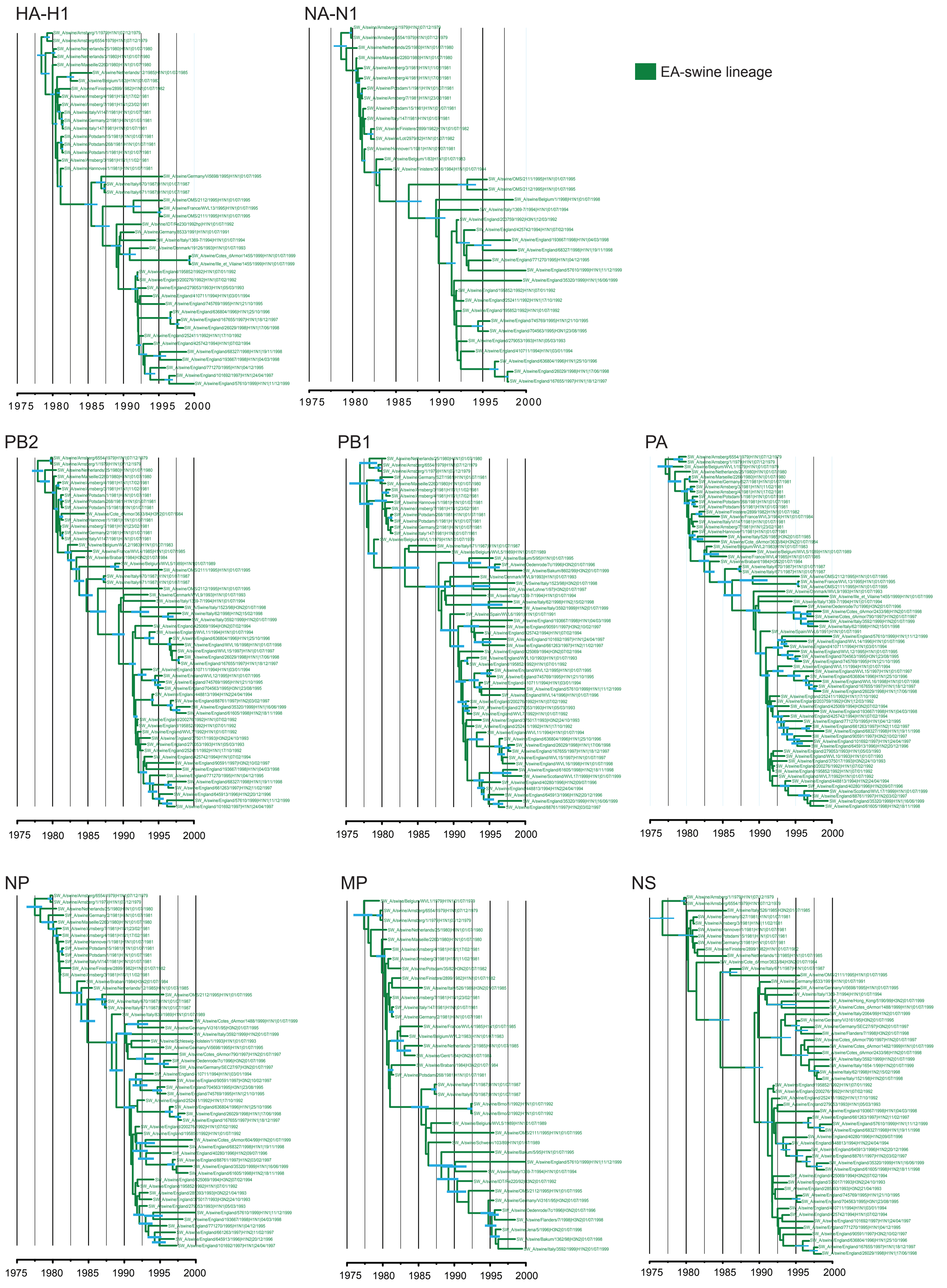
HA-H1
NA-N1
EA-swine lineage
PB2
PB1
PA
NP
MP
NS
1975
1980
1985
1990
1995
2000

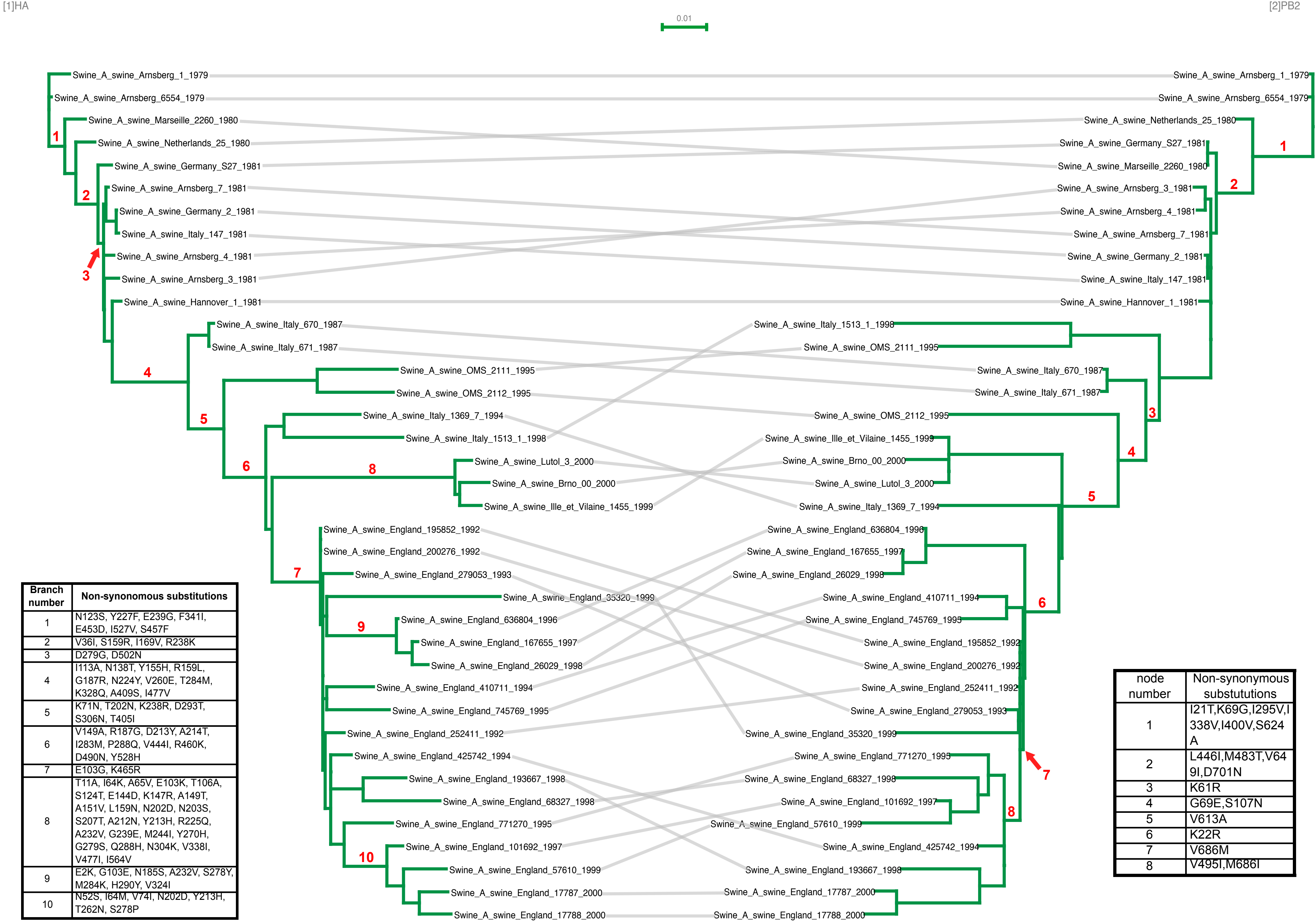

Branch number	Non-synonymous substitutions
1	N123S, Y227F, E239G, F341I, E453D, I527V, S457F
2	V36I, S159R, I169V, R238K
3	D279G, D502N
4	I113A, N138T, Y155H, R159L, G187R, N224Y, V260E, T284M, K328Q, A409S, I477V
5	K71N, T202N, K238R, D293T, S306N, T405I
6	V149A, R187G, D213Y, A214T, I283M, P288Q, V444I, R460K, D490N, Y528H
7	E103G, K465R
8	T11A, I64K, A65V, E103K, T106A, S124T, E144D, K147R, A149T, A151V, L159N, N202D, N203S, S207T, A212N, Y213H, R225Q, A232V, G239E, M244I, Y270H, G279S, Q288H, N304K, V338I, V477I, I564V
9	E2K, G103E, N185S, A232V, S278Y, M284K, H290Y, V324I
10	N52S, I64M, V74I, N202D, Y213H, T262N, S278P

node number	Non-synonymous substitutions
1	I21T,K69G,I295V,I338V,I400V,S624A
2	L446I,M483T,V649I,D701N
3	K61R
4	G69E,S107N
5	V613A
6	K22R
7	V686M
8	V495I,M686I

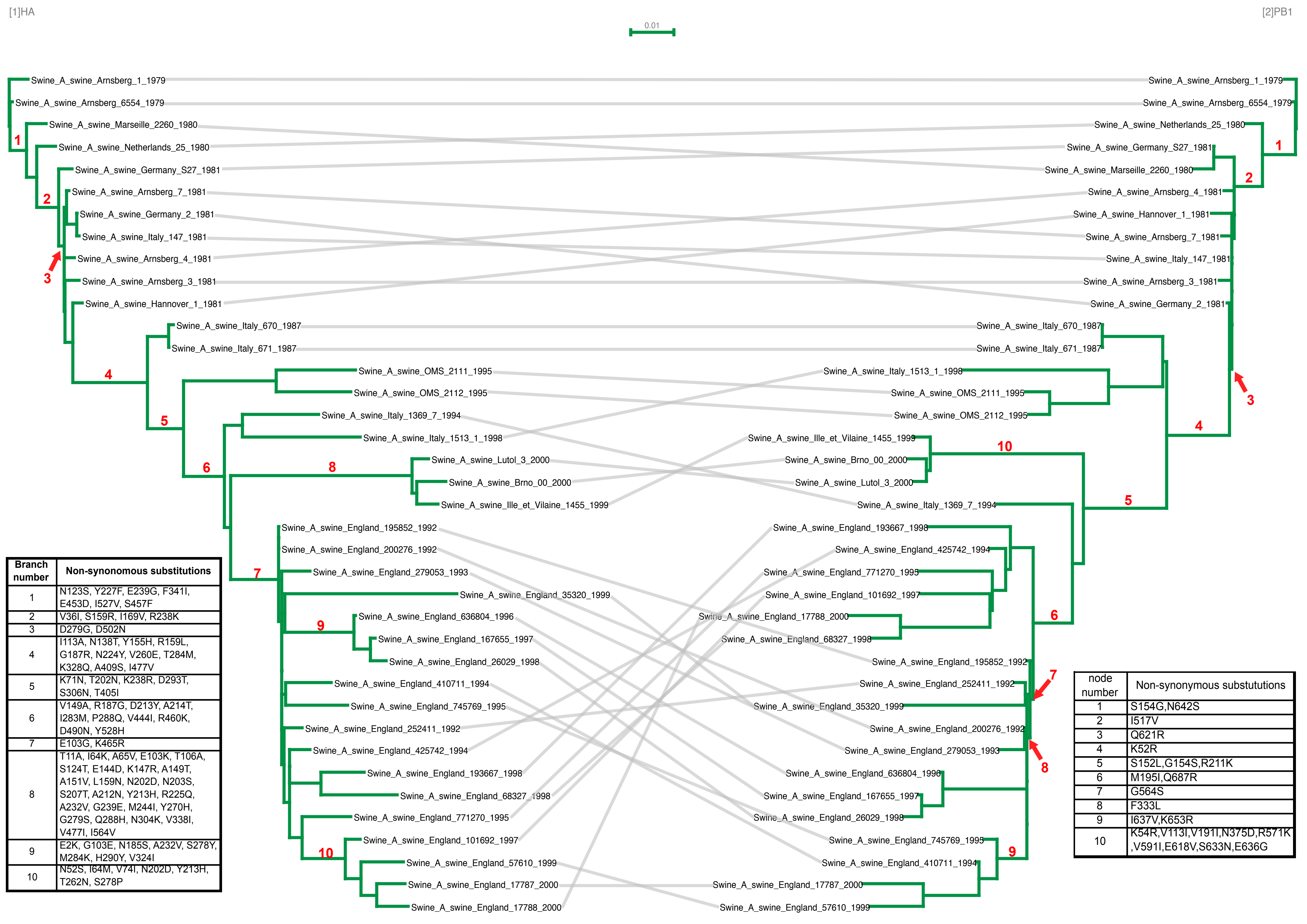

Branch number	Non-synonomous substitutions
1	N123S, Y227F, E239G, F341I, E453D, I527V, S457F
2	V36I, S159R, I169V, R238K
3	D279G, D502N
4	I113A, N138T, Y155H, R159L, G187R, N224Y, V260E, T284M, K328Q, A409S, I477V
5	K71N, T202N, K238R, D293T, S306N, T405I
6	V149A, R187G, D213Y, A214T, I283M, P288Q, V444I, R460K, D490N, Y528H
7	E103G, K465R
8	T11A, I64K, A65V, E103K, T106A, S124T, E144D, K147R, A149T, A151V, L159N, N202D, N203S, S207T, A212N, Y213H, R225Q, A232V, G239E, M244I, Y270H, G279S, Q288H, N304K, V338I, V477I, I564V
9	E2K, G103E, N185S, A232V, S278Y, M284K, H290Y, V324I
10	N52S, I64M, V74I, N202D, Y213H, T262N, S278P

node number	Non-synonymous substututions
1	S154G,N642S
2	I517V
3	Q621R
4	K52R
5	S152L,G154S,R211K
6	M195I,Q687R
7	G564S
8	F333L
9	I637V,K653R
10	K54R,V113I,V191I,N375D,R571K ,V591I,E618V,S633N,E636G

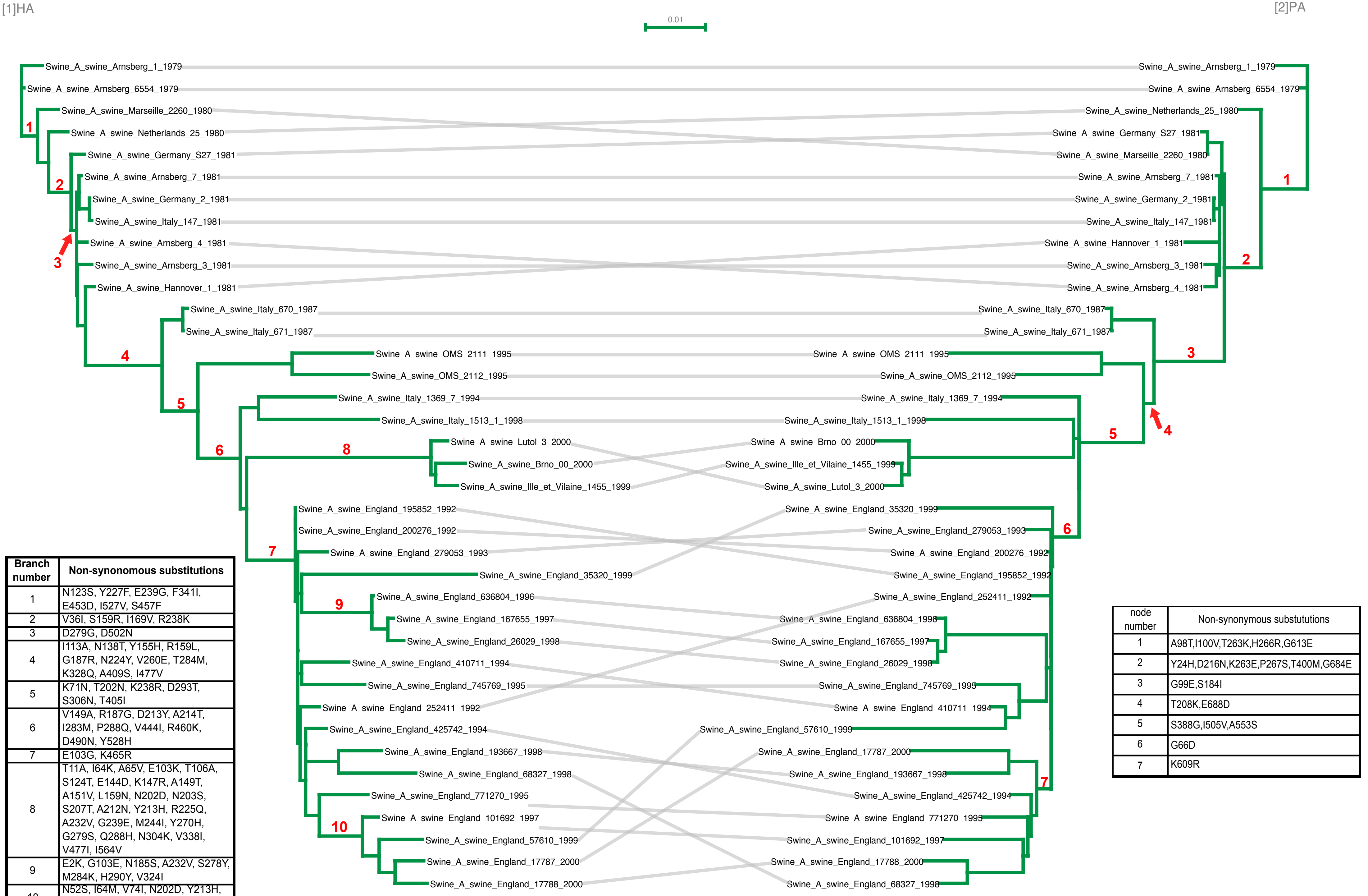

Branch number	Non-synonomous substitutions
1	N123S, Y227F, E239G, F341I, E453D, I527V, S457F
2	V36I, S159R, I169V, R238K
3	D279G, D502N
4	I113A, N138T, Y155H, R159L, G187R, N224Y, V260E, T284M, K328Q, A409S, I477V
5	K71N, T202N, K238R, D293T, S306N, T405I
6	V149A, R187G, D213Y, A214T, I283M, P288Q, V444I, R460K, D490N, Y528H
7	E103G, K465R
8	T11A, I64K, A65V, E103K, T106A, S124T, E144D, K147R, A149T, A151V, L159N, N202D, N203S, S207T, A212N, Y213H, R225Q, A232V, G239E, M244I, Y270H, G279S, Q288H, N304K, V338I, V477I, I564V
9	E2K, G103E, N185S, A232V, S278Y, M284K, H290Y, V324I
10	N52S, I64M, V74I, N202D, Y213H, T262N, S278P

node number	Non-synonymous substutions
1	A98T,I100V,T263K,H266R,G613E
2	Y24H,D216N,K263E,P267S,T400M,G684E
3	G99E,S184I
4	T208K,E688D
5	S388G,I505V,A553S
6	G66D
7	K609R

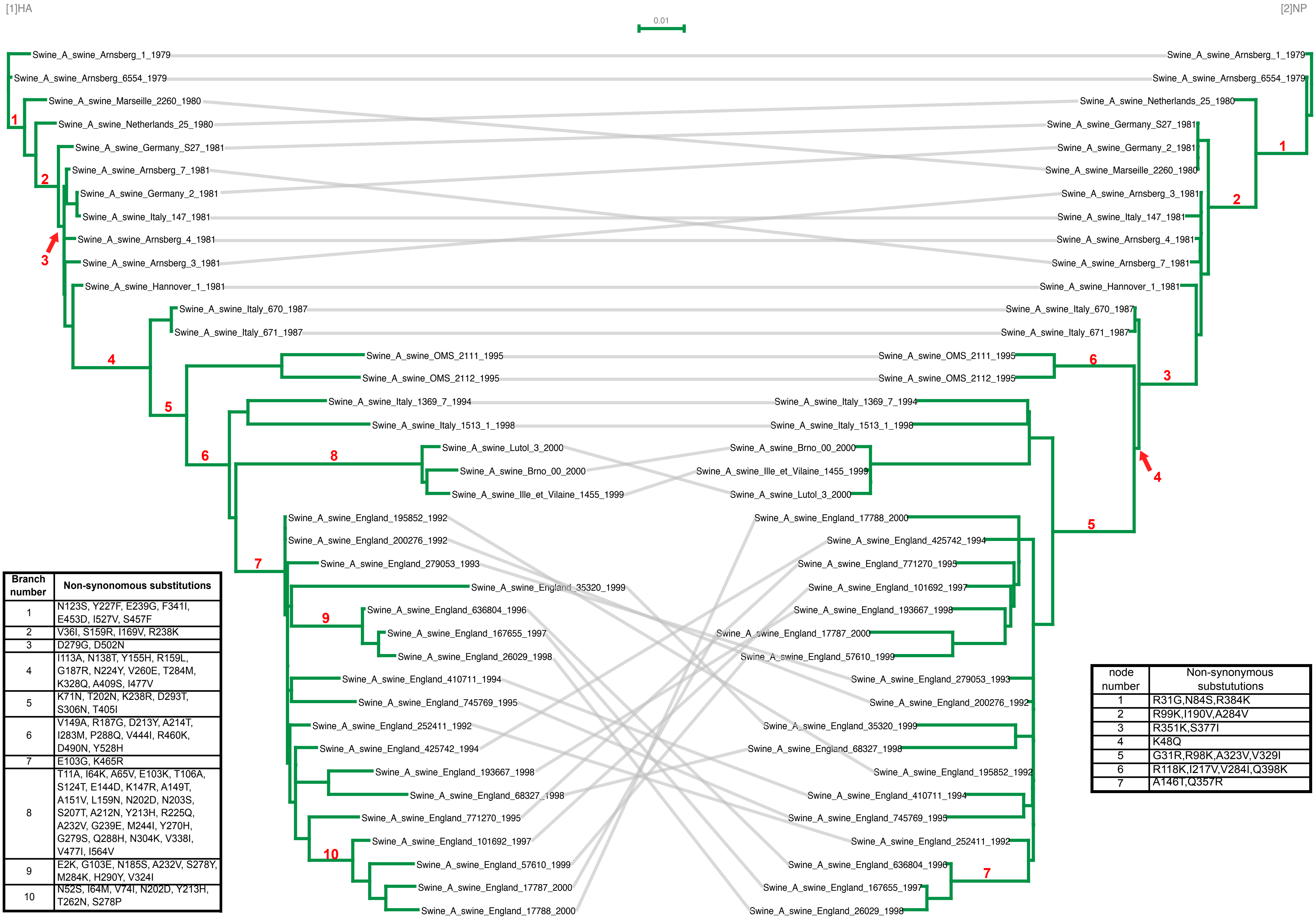

Branch number	Non-synonomous substitutions
1	N123S, Y227F, E239G, F341I, E453D, I527V, S457F
2	V36I, S159R, I169V, R238K
3	D279G, D502N
4	I113A, N138T, Y155H, R159L, G187R, N224Y, V260E, T284M, K328Q, A409S, I477V
5	K71N, T202N, K238R, D293T, S306N, T405I
6	V149A, R187G, D213Y, A214T, I283M, P288Q, V444I, R460K, D490N, Y528H
7	E103G, K465R
8	T11A, I64K, A65V, E103K, T106A, S124T, E144D, K147R, A149T, A151V, L159N, N202D, N203S, S207T, A212N, Y213H, R225Q, A232V, G239E, M244I, Y270H, G279S, Q288H, N304K, V338I, V477I, I564V
9	E2K, G103E, N185S, A232V, S278Y, M284K, H290Y, V324I
10	N52S, I64M, V74I, N202D, Y213H, T262N, S278P

node number	Non-synonymous substitutions
1	R31G,N84S,R384K
2	R99K,I190V,A284V
3	R351K,S377I
4	K48Q
5	G31R,R98K,A323V,V329I
6	R118K,I217V,V284I,Q398K
7	A146T,Q357R

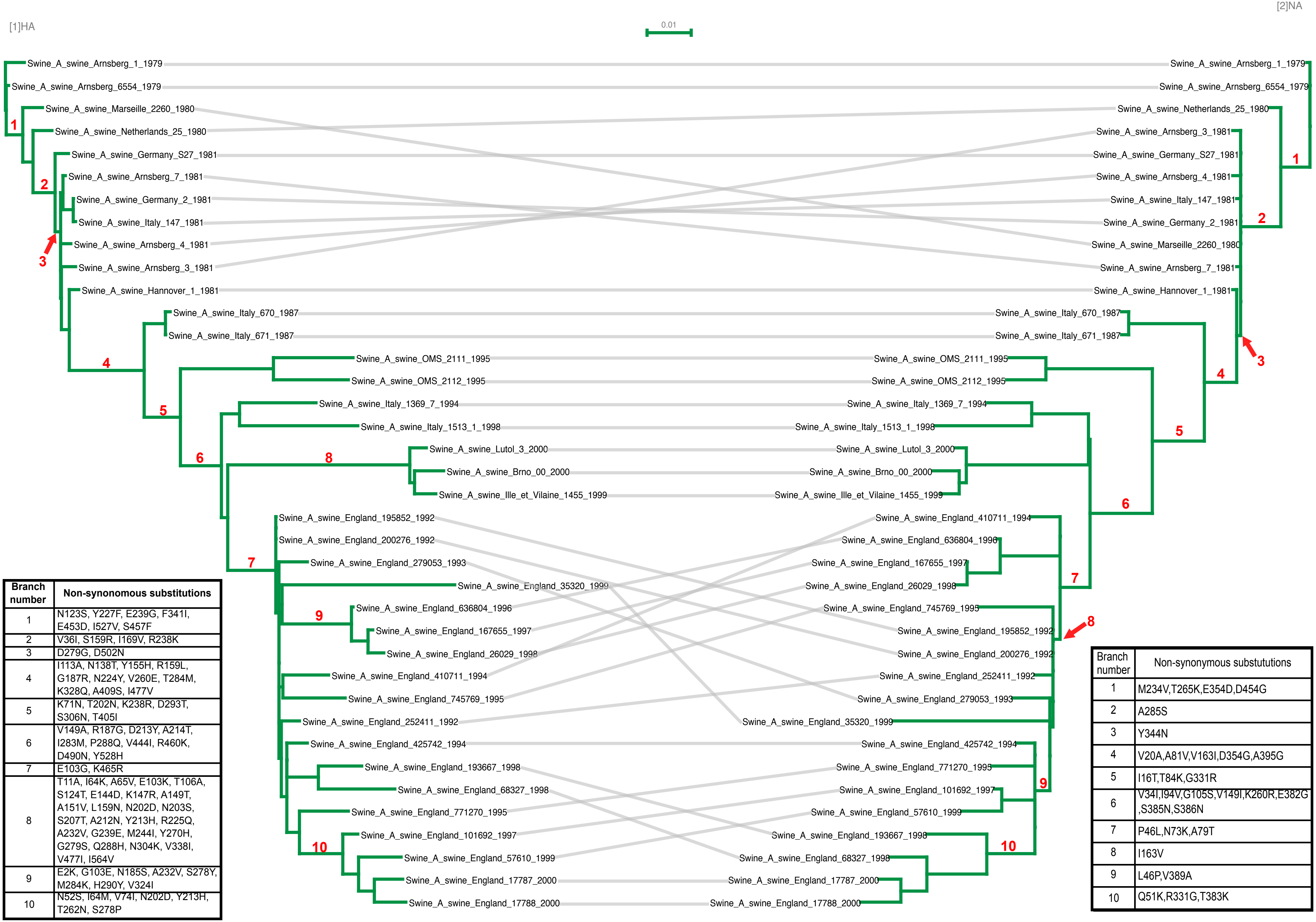

Branch number	Non-synonomous substitutions
1	N123S, Y227F, E239G, F341I, E453D, I527V, S457F
2	V36I, S159R, I169V, R238K
3	D279G, D502N
4	I113A, N138T, Y155H, R159L, G187R, N224Y, V260E, T284M, K328Q, A409S, I477V
5	K71N, T202N, K238R, D293T, S306N, T405I
6	V149A, R187G, D213Y, A214T, I283M, P288Q, V444I, R460K, D490N, Y528H
7	E103G, K465R
8	T11A, I64K, A65V, E103K, T106A, S124T, E144D, K147R, A149T, A151V, L159N, N202D, N203S, S207T, A212N, Y213H, R225Q, A232V, G239E, M244I, Y270H, G279S, Q288H, N304K, V338I, V477I, I564V
9	E2K, G103E, N185S, A232V, S278Y, M284K, H290Y, V324I
10	N52S, I64M, V74I, N202D, Y213H, T262N, S278P

Branch number	Non-synonymous substututions
1	M234V,T265K,E354D,D454G
2	A285S
3	Y344N
4	V20A,A81V,V163I,D354G,A395G
5	I16T,T84K,G331R
6	V34I,I94V,G105S,V149I,K260R,E382G ,S385N,S386N
7	P46L,N73K,A79T
8	I163V
9	L46P,V389A
10	Q51K,R331G,T383K

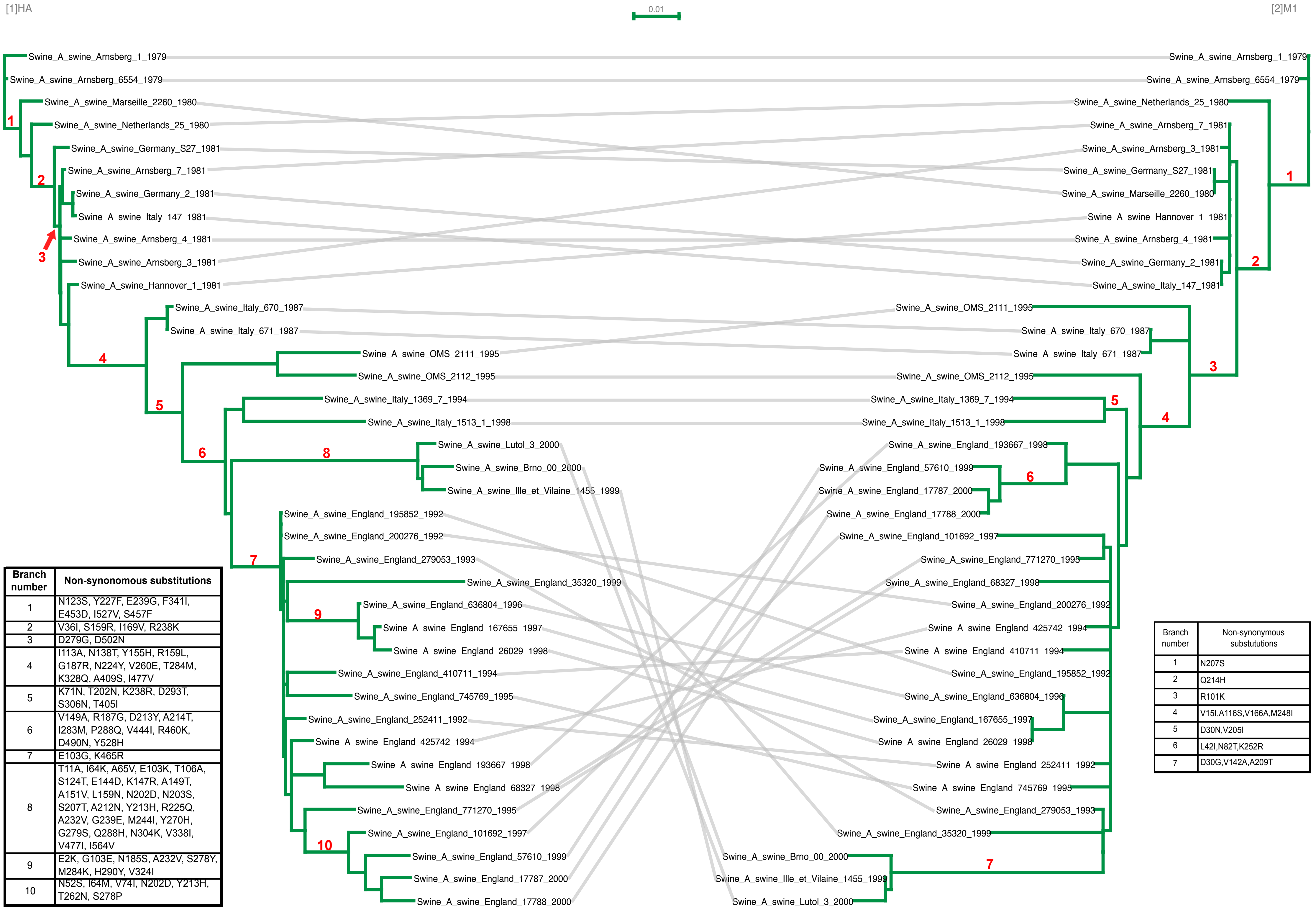

Branch number	Non-synonymous substitutions
1	N123S, Y227F, E239G, F341I, E453D, I527V, S457F
2	V36I, S159R, I169V, R238K
3	D279G, D502N
4	I113A, N138T, Y155H, R159L, G187R, N224Y, V260E, T284M, K328Q, A409S, I477V
5	K71N, T202N, K238R, D293T, S306N, T405I
6	V149A, R187G, D213Y, A214T, I283M, P288Q, V444I, R460K, D490N, Y528H
7	E103G, K465R
8	T11A, I64K, A65V, E103K, T106A, S124T, E144D, K147R, A149T, A151V, L159N, N202D, N203S, S207T, A212N, Y213H, R225Q, A232V, G239E, M244I, Y270H, G279S, Q288H, N304K, V338I, V477I, I564V
9	E2K, G103E, N185S, A232V, S278Y, M284K, H290Y, V324I
10	N52S, I64M, V74I, N202D, Y213H, T262N, S278P

Branch number	Non-synonymous substitutions
1	N207S
2	Q214H
3	R101K
4	V15I,A116S,V166A,M248I
5	D30N,V205I
6	L42I,N82T,K252R
7	D30G,V142A,A209T

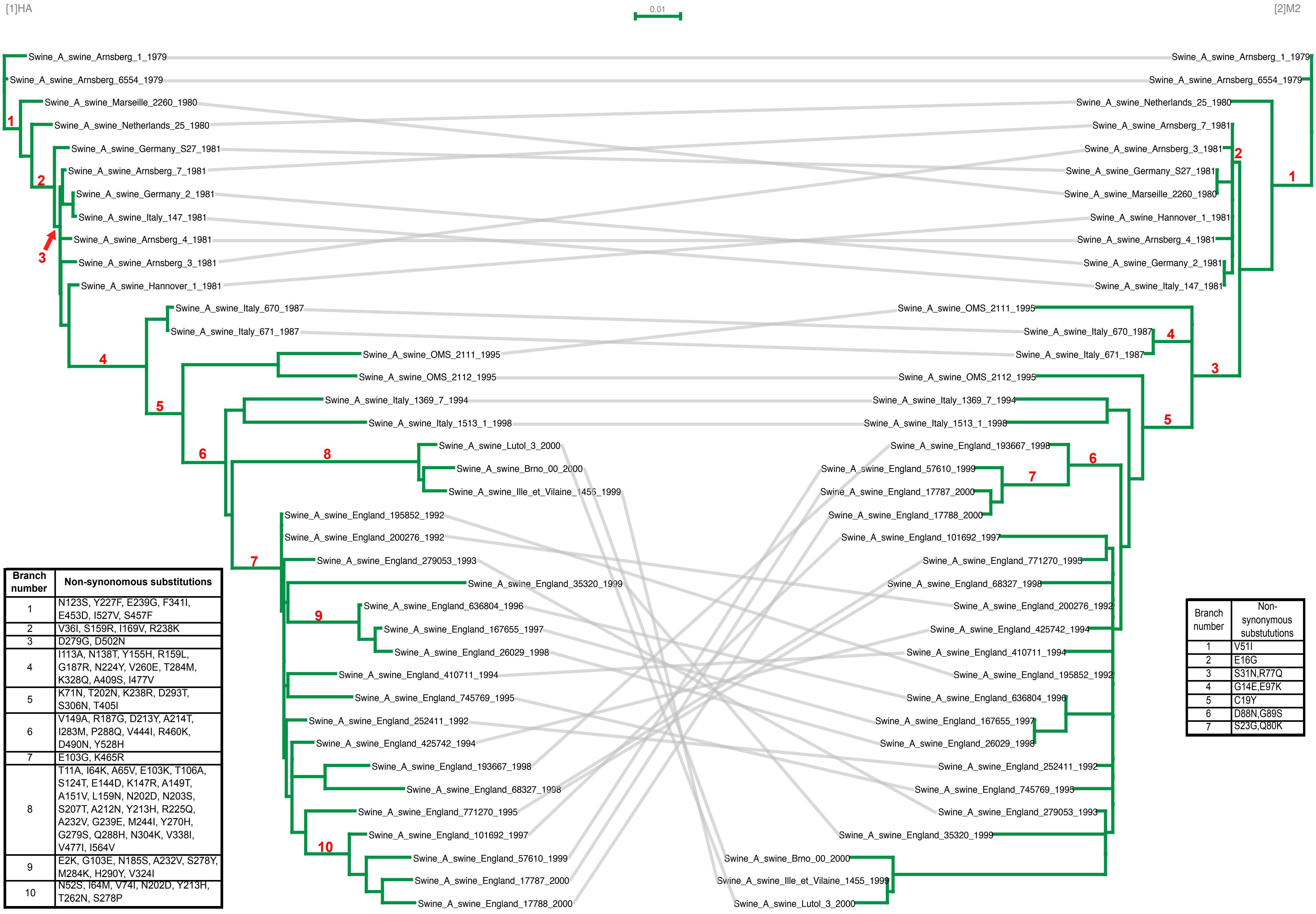

Branch number	Non-synonomous substitutions
1	N123S, Y227F, E239G, F341I, E453D, I527V, S457F
2	V36I, S159R, I169V, R238K
3	D279G, D502N
4	I113A, N138T, Y155H, R159L, G187R, N224Y, V260E, T284M, K328Q, A409S, I477V
5	K71N, T202N, K238R, D293T, S306N, T405I
6	V149A, R187G, D213Y, A214T, I283M, P288Q, V444I, R460K, D490N, Y528H
7	E103G, K465R
8	T11A, I64K, A65V, E103K, T106A, S124T, E144D, K147R, A149T, A151V, L159N, N202D, N203S, S207T, A212N, Y213H, R225Q, A232V, G239E, M244I, Y270H, G279S, Q288H, N304K, V338I, V477I, I564V
9	E2K, G103E, N185S, A232V, S278Y, M284K, H290Y, V324I
10	N52S, I64M, V74I, N202D, Y213H, T262N, S278P

Branch number	Non-synonymous substitutions
1	V51I
2	E16G
3	S31N,R77Q
4	G14E,E97K
5	C19Y
6	D88N,G89S
7	S23G,Q80K

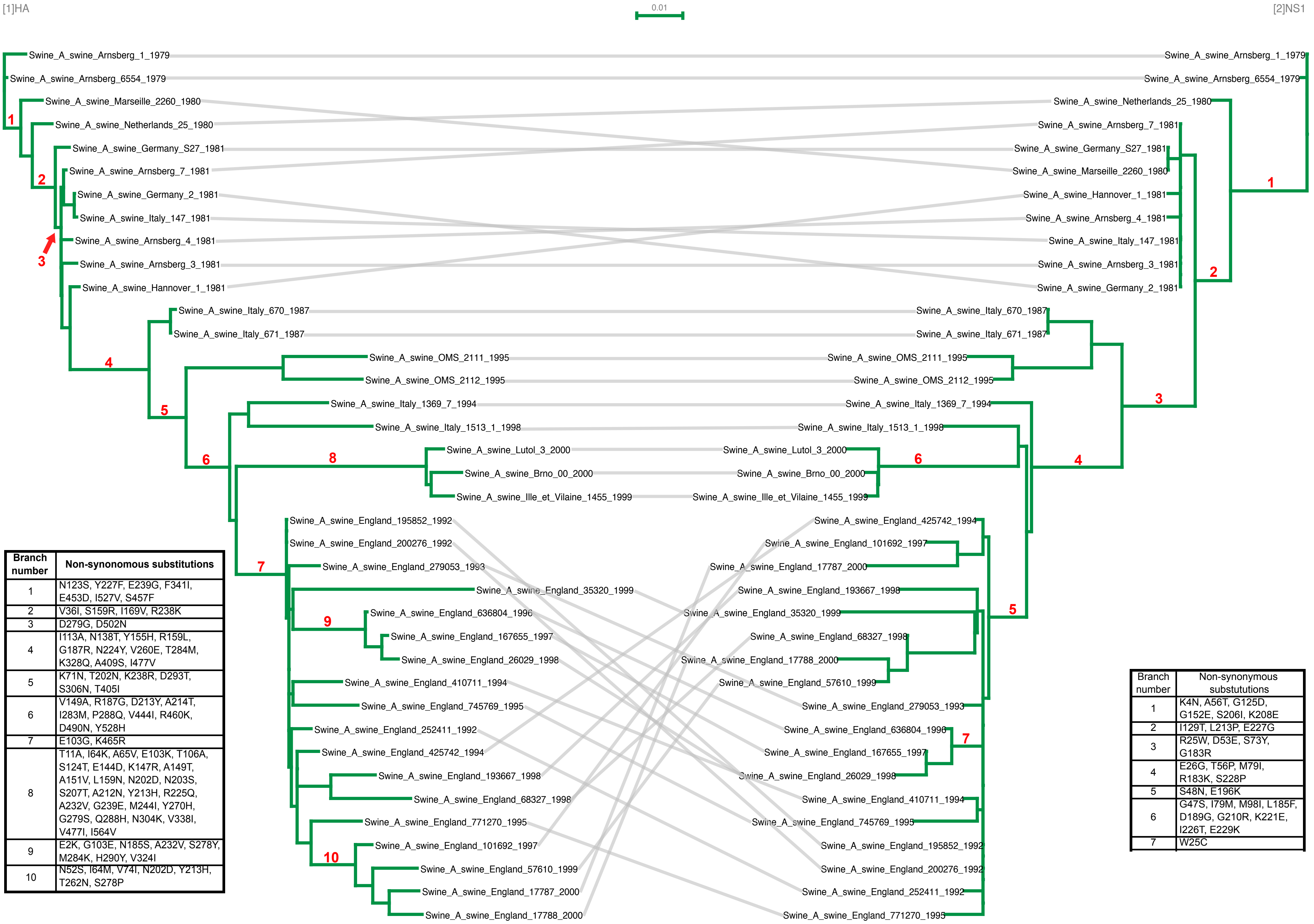

Branch number	Non-synonomous substitutions
1	N123S, Y227F, E239G, F341I, E453D, I527V, S457F
2	V36I, S159R, I169V, R238K
3	D279G, D502N
4	I113A, N138T, Y155H, R159L, G187R, N224Y, V260E, T284M, K328Q, A409S, I477V
5	K71N, T202N, K238R, D293T, S306N, T405I
6	V149A, R187G, D213Y, A214T, I283M, P288Q, V444I, R460K, D490N, Y528H
7	E103G, K465R
8	T11A, I64K, A65V, E103K, T106A, S124T, E144D, K147R, A149T, A151V, L159N, N202D, N203S, S207T, A212N, Y213H, R225Q, A232V, G239E, M244I, Y270H, G279S, Q288H, N304K, V338I, V477I, I564V
9	E2K, G103E, N185S, A232V, S278Y, M284K, H290Y, V324I
10	N52S, I64M, V74I, N202D, Y213H, T262N, S278P

Branch number	Non-synonymous substitutions
1	K4N, A56T, G125D, G152E, S206I, K208E
2	I129T, L213P, E227G
3	R25W, D53E, S73Y, G183R
4	E26G, T56P, M79I, R183K, S228P
5	S48N, E196K
6	G47S, I79M, M98I, L185F, D189G, G210R, K221E, I226T, E229K
7	W25C

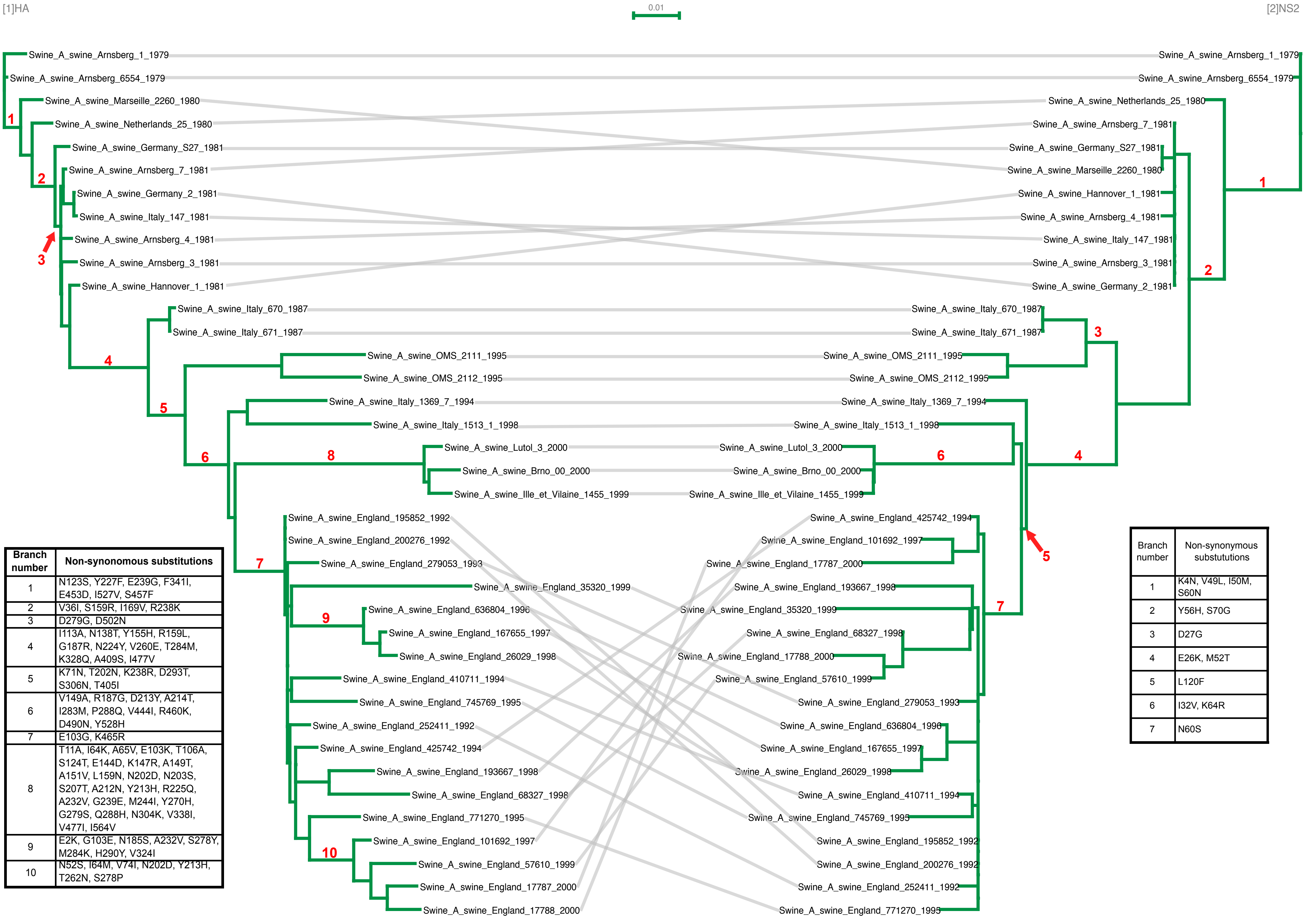

Branch number	Non-synonymous substitutions
1	N123S, Y227F, E239G, F341I, E453D, I527V, S457F
2	V36I, S159R, I169V, R238K
3	D279G, D502N
4	I113A, N138T, Y155H, R159L, G187R, N224Y, V260E, T284M, K328Q, A409S, I477V
5	K71N, T202N, K238R, D293T, S306N, T405I
6	V149A, R187G, D213Y, A214T, I283M, P288Q, V444I, R460K, D490N, Y528H
7	E103G, K465R
8	T11A, I64K, A65V, E103K, T106A, S124T, E144D, K147R, A149T, A151V, L159N, N202D, N203S, S207T, A212N, Y213H, R225Q, A232V, G239E, M244I, Y270H, G279S, Q288H, N304K, V338I, V477I, I564V
9	E2K, G103E, N185S, A232V, S278Y, M284K, H290Y, V324I
10	N52S, I64M, V74I, N202D, Y213H, T262N, S278P

Branch number	Non-synonymous substitutions
1	K4N, V49L, I50M, S60N
2	Y56H, S70G
3	D27G
4	E26K, M52T
5	L120F
6	I32V, K64R
7	N60S
